# Molecular Dynamics of the Intrinsically Disordered Protein COR15A—A Force Field Validation on Structure and Dynamics

**DOI:** 10.1021/acs.jctc.5c00854

**Published:** 2025-09-04

**Authors:** Tobias Rindfleisch, Ricky Nencini, O. H. Samuli Ollila, Dirk Walther, Markus S. Miettinen, Anja Thalhammer

**Affiliations:** † Computational Biology Unit, Department of Informatics, 1658 University of Bergen, 5008 Bergen, Norway; ‡ Department of Chemistry, University of Bergen, 5007 Bergen, Norway; § Physical Biochemistry, 26583 University of Potsdam, 14476 Potsdam, Germany; ∥ Max-Planck Institute of Molecular Plant Physiology, 14476 Potsdam, Germany; ⊥ Institute of Biotechnology, 3835 University of Helsinki, 00014 Helsinki, Finland; # Division of Pharmaceutical Biosciences, Faculty of Pharmacy, University of Helsinki, 00790 Helsinki, Finland; ¶ VTT Technical Research Centre of Finland, 02044 Espoo, Finland; ∇ Department of Theory and Bio-Systems, Max Planck Institute of Colloids and Interfaces, 14476 Potsdam, Germany

## Abstract

Intrinsically disordered proteins (IDPs) pose a challenge for structural characterization, as experimental methods lack the subnanometer/subnanosecond resolution to capture their dynamic conformational ensembles. Molecular dynamics (MD) simulations can, in principle, provide this information, but for the simulation of IDPs, dedicated protein and water force fields are needed, as traditional MD models for folded proteins prove inadequate for IDPs. Substantial effort was invested to develop IDP-specific force fields, but their performance in describing IDPs that undergo conformational changes—such as those induced by molecular partner binding or changes in solution environment—remains underexplored. In this study, we investigated the ability of 20 MD models to accurately simulate structural and dynamic aspects of COR15A, an IDP just on the verge of folding, with a particular focus on their ability to capture subtle structural differences. We employ a two-step approach: (i) validation of short 200 ns simulations against small-angle X-ray scattering (SAXS) data and (ii) detailed evaluation of the six best-performing MD models through extended 1.2 μs MD simulations against nuclear magnetic resonance (NMR) data, including a single-point mutant with slightly increased helicity. Only DES-amber and ff99SBws capture helicity differences between wild-type and mutant, but ff99SBws overestimates helicity. Notably, only DES-amber adequately reproduces the COR15A dynamics, as assessed by NMR relaxation times at two different magnetic field strengths. Among the tested force fields, DES-amber emerges as the best MD model for the simulation of COR15A. Its application provides insights into its dynamic conformational landscape, albeit not perfectly reproducing all experimental data. Our study highlights the need for rigorous force field validation for IDPs and identifies remaining discrepancies in need of further force-field development.

## Graphical abstract

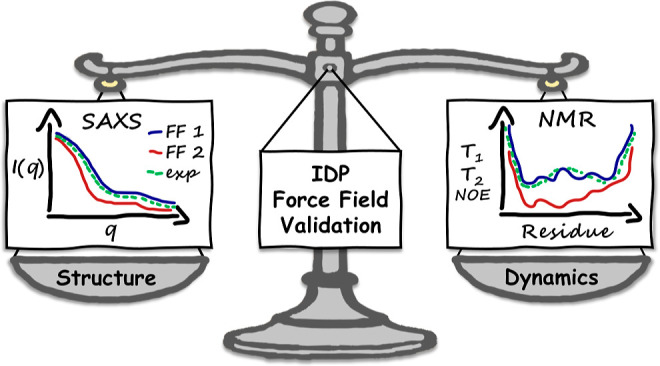

## 1 Introduction

It is now firmly established that many proteins do not fold into single conformations under physiological conditions.^1^ In contrast to the well-established notion that protein function is tied to precisely folded structures, the functionality of these proteins appears to depend on their rich conformational ensembles. These so-called intrinsically disordered proteins (IDPs) fulfill important and specific functions, e.g., in cellular signaling and regulation of metabolic processes.^2,3^ IDPs are enriched in charged and hydrophilic amino acids, while hydrophobic residues are underrepresented, which impedes the establishment of a hydrophobic core necessary to form stably folded structures.^4,5^ In addition to fully disordered proteins, intrinsically disordered regions (IDRs) are often present within otherwise well-structured proteins, and the term IDP also commonly includes proteins that contain IDRs.^6^ Some IDPs are fully functional in the disordered state; for others, interaction with binding partners is required, which often, but not always, results in a significant structural rearrangement of the IDP.^3^ Protein disorder is found in all kingdoms of life, but is most abundant in eukaryotes:^7^ Bioinformatics studies indicate that roughly 25% of all eukaryotic proteins are fully unstructured,^8^ and 40% contain IDRs.^9^

Molecular motions within an IDP ensemble, occurring on picosecond to millisecond time scales,^10^ present a significant challenge for experimental techniques, as these often capture time- and ensemble-based averages, not individual conformational states. This limits the investigation of specific ensemble characteristics, such as the conformational distribution, the presence of transiently populated states, and dynamics of the transitions between them. A widely established alternative to explore protein dynamics at subnanometer–subnanosecond resolution is molecular dynamics (MD) simulation,^11,12^ which has the power to bridge the gaps in experimental data and provide detailed atomic insights into IDP dynamics.^13^

However, adequately describing an IDP’s ensemble of continuously fluctuating conformations is challenging also in MD,^14,15^ because most MD force fields have been developed in the context of folded, globular proteins.^16,17^ They tend to overestimate intramolecular attraction, and thus the compactness of IDPs is generally overestimated.^16,18,19^

Consequently, efforts have been made to develop IDP-specific force fields. The main focus has been on modifying protein–water (Lennard-Jones) interactions to increase protein hydration. With an additional adjustment of the water model to improve solvent representation, a better agreement with small-angle X-ray scattering (SAXS) and Förster resonance energy transfer (FRET) was reached.^18,20^ Further optimization was accomplished by modifications of torsion parameters;^20^ in particular, the dihedrals defining the rotation of side-chain methyl groups (C^γ^) were improved against nuclear magnetic resonance (NMR) data.^21,22^ An alternative approach to solve the problem of insufficient hydration was taken by the CUFIX force fields: decreasing the protein–protein interaction, which allows for the continued use of established water models such as TIP3P.^23^

To use MD simulations to understand the complex structure–function relationships of IDPs, combinations of protein and water force fields that robustly capture the dynamic conformational ensembles are needed. They must perform well for IDPs with very different sequences and conformational properties, including the more folded conformations favored by binding partners or specific solution environments.

A group of IDPs particularly sensitive to the latter are the late embryogenesis abundant (LEA) proteins that accumulate in plants and several anhydrobiotic nonplant organisms during developmental stages or abiotic stresses involving cellular dehydration. How LEA proteins improve the organismal tolerance to water limitation upon exposure to freezing, desiccation, drought, high salinity, high osmolarity, and molecular crowding^24^ is not yet fully understood at the molecular level, but is potentially rooted in their stabilizing effect on membranes and/or proteins (reviewed in Hernández-Sánchez et al.^24^). Most experimentally investigated LEA proteins are almost fully disordered in aqueous media, but sample more compact folded conformations in response to various molecular triggers.^24^ This is reflected in disorder predictions: Most LEA proteins are predicted fully disordered throughout their sequences, but with per-residue disorder propensities around 50%, indicative of protein sequences that appear just on the verge of disorder^25^ (see, e.g., Figure S1).

This property renders LEA proteins excellent candidates for systematically testing the robustness of IDP force fields. Here, we focus on one of the best characterized LEA proteins, COld Regulated (COR)­15A, from the model plant *Arabidopsis thaliana*. Like many LEA proteins, COR15A is almost completely disordered in its hydrated state and undergoes a fully reversible coil–helix transition in response to partial and complete dehydration.^26–28^

We present systematic comparative analyses against experimental scattering and NMR data for a wide set of MD models—protein and water force fields—regarding their suitability for simulations of the hydrated state of the folding-prone COR15A wild-type (WT). We further assess its point mutant G68A, experimentally characterized by slightly higher helicity in the hydrated state and a more distinct coil–helix transition.

## 2 Results and Discussion

### 2.1 Initial Screening of MD Models for COR15A WT

Ten independent initial structures were prepared using the OPLS-AA force field; see Experimental Section. For each of these 10 representatives of the COR15A WT conformational ensemble, 200 ns simulations were performed using each of the 20 protein and water force field combinations listed in Table 1. During equilibration, the total potential energy (*E*_pot_) and root-mean-square deviation (RMSD) from the initial structures were monitored as a function of time (Figure S2). The RMSD indicated that the simulations reached characteristic conformational sampling within 120 ns for all 20 MD models. Consequently, the initial 120 ns of these simulations were treated as an extended equilibration period, with all structure-related analyses performed exclusively on the subsequent 80 ns production runs.

#### 2.1.1 Radius of Gyration

Figure 1 compares the distributions of radius of gyration (*R*_G_) in all 20 different MD models (Figure S3) against the *R*_G_ experimentally determined by SAXS.^29^

**Figure 1:**
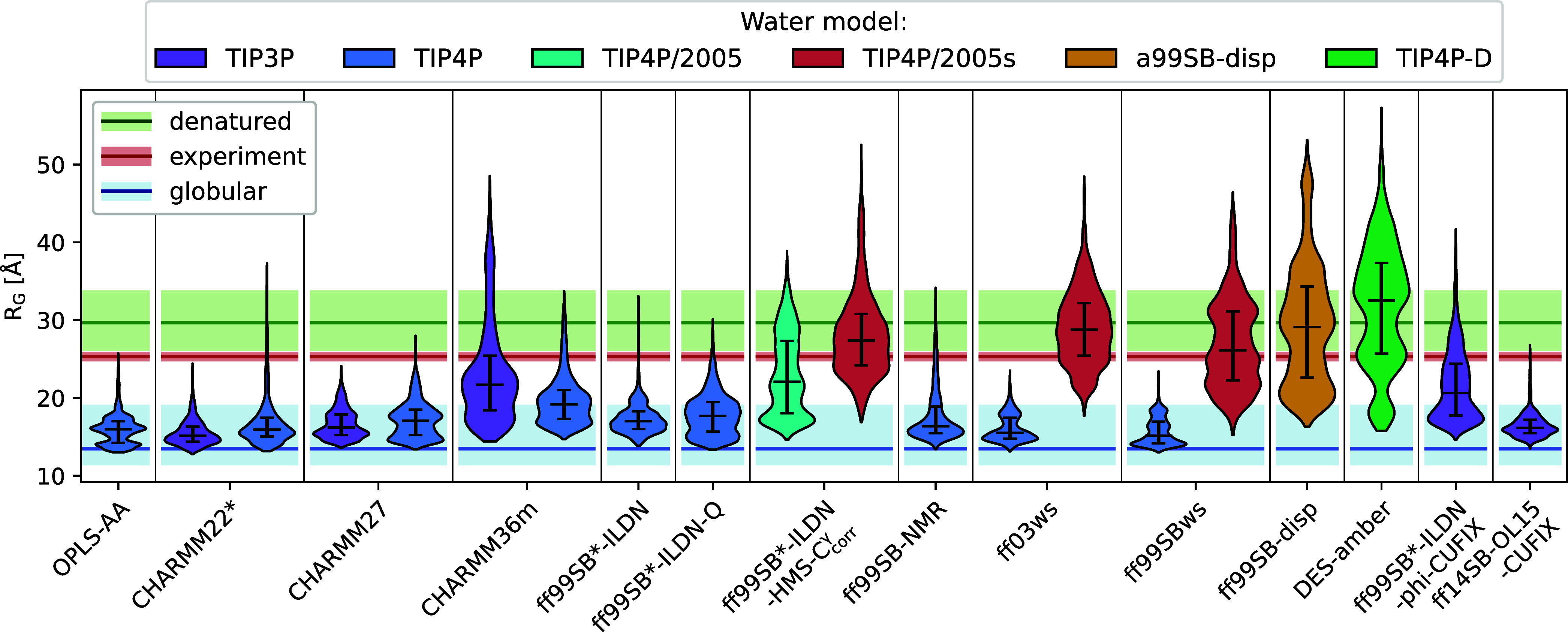
*R*_G_ for all 20 tested MD models (see Table 1 for simulation details). Each violin plot shows the *R*_G_ distribution across all conformations sampled in the 10 equilibrated 80 ns replicate simulations. Violins are color-coded to indicate the water model, long dashes represent the median, and error bars represent the 25% and 75% quartiles. The red horizontal line shows the experimental reference determined by SAXS;^29^ in addition, the blue line shows the *R*_G_ approximated from scaling laws for globular^30^ (blue line) and denatured^31^ (green line) proteins with molecular weights identical to COR15A WT. The estimated error is visualized in lighter color.

Water force fields TIP3P and TIP4P are seen to result (with the exception of CHARMM36m and ff99SB*-ILDN phi-CUFIX in conjunction with TIP3P) in *R*_G_ distributions typical for globular well-folded proteins, 12 Å < *R*_G_ < 18 Å, overestimating the compactness of COR15A WT (Figure 1). This indicates, despite the positive skewness (a longer tail toward higher *R*_G_, Figure S4A,B), that TIP3P and TIP4P are generally less suited for IDPs: Their predicted *R*_G_ distributions are more common for globular or collapsed proteins.^16,32^

The Amber force fields in conjunction with their associated water models are generally better suited to describe experimentally determined *R*_G_. Specifically, ff03ws, ff99SBws, ff99SB-disp, DES-amber, and ff99SB*-ILDN-HMS-C_corr_^γ^, when combined with an appropriate water model, succeeded in modeling the extended structure of COR15A WT by capturing, within error bars, the experimental *R*_G_ with their broad *R*_G_ distributions ranging from very compact to highly extended conformations (Figure S4C).

#### 2.1.2 Complete SAXS Scattering Profiles

Figure 2 presents a comparison of the computed and experimental SAXS profiles for OPLS-AA (previously used in COR15A MD studies) and the eight MD models with the most realistic *R*_G_, as shown in Figure 1. A SAXS profile provides a wealth of information: The low-*q* Guinier region (for unfolded proteins: *qR*_*G*_ < 1.1 ^33^) on object size (*R*_G_) and molecular mass, the middle-*q* Fourier region on shape and flexibility, and the high-*q* Porod region on surface properties.^34–36^

**Figure 2:**
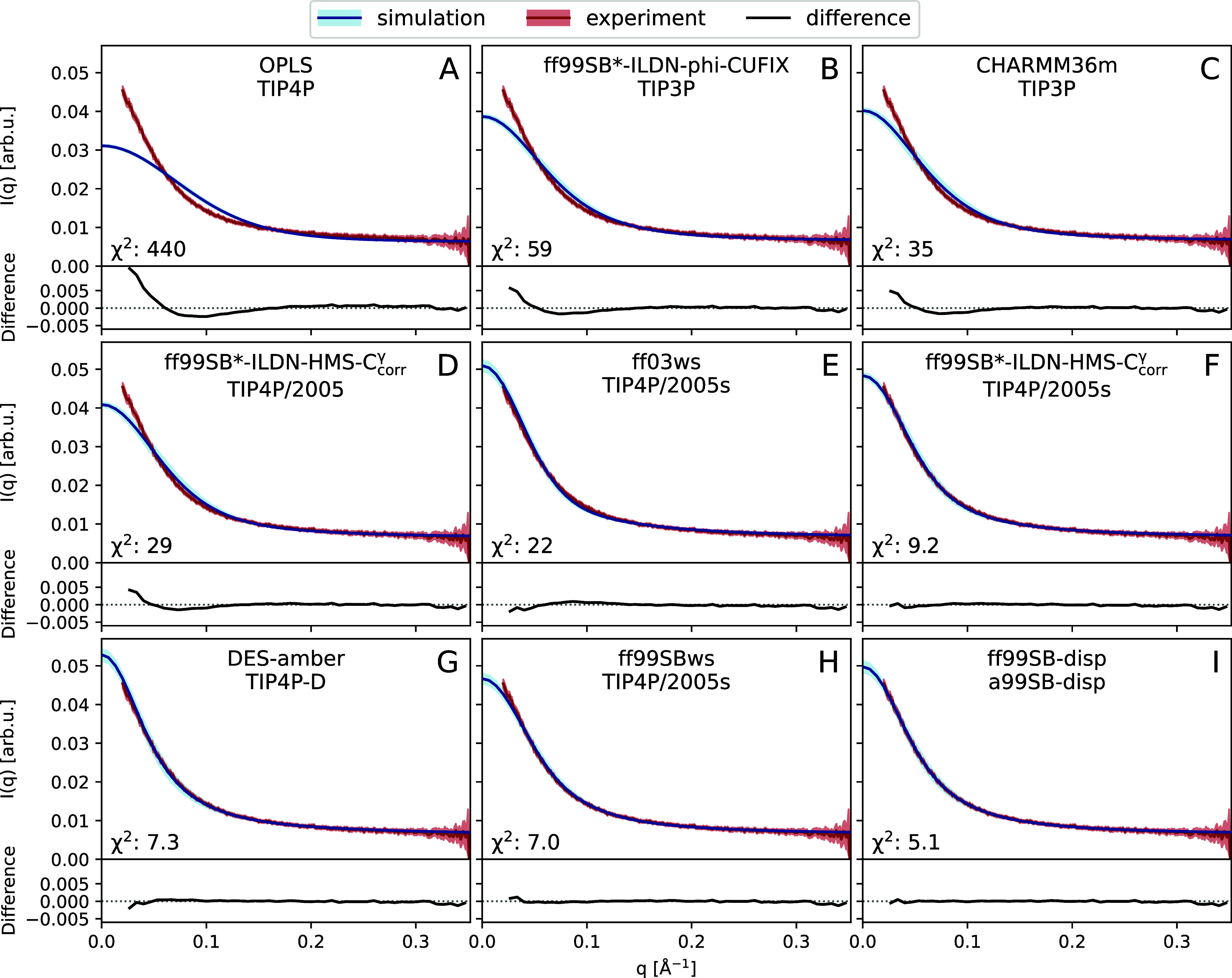
COR15A WT SAXS profiles for nine selected MD models (A–I), each computed from *n* = 10 independent simulations (blue), compared against experiment^29^ (red). See Figure S5 for profiles of all 20 tested MD models and Figure S7 for double-logarithmic presentation. Standard errors of the mean (SEM) for the computed profiles and the experimental errors are indicated in lighter shades. The χ^2^ is between experiment and simulation; the lower panel in each subplot shows the difference (experiment minus simulation).

Beyond the Guinier region (*q* ≥ 0.15 Å^–1^), difference plots illustrate a fair to very good agreement for all simulations (Figures 2 and S5), which concurs with the almost complete disorder of COR15A WT.^29^ This agrees also with the Kratky plots,^34,35^ in which globular proteins show a clear bell-shape followed by an asymptotic zero plateau, and disordered proteins a monotonic increase at high *q*;^37^ Figure S6 shows that in all MD simulations COR15A WT was in the disordered state, and the similarity to experimental data is high. Interestingly, a consistent separation based on the water model is discernible with all waters originally developed in the IDP context, demonstrating excellent agreement with the experimental Kratky profile.

As the initial slope of a scattering profile correlates with *R*_G_,^34^ the low *R*_G_ of most MD models (Figure 1) shows as an obvious divergence from the experiment in the Guinier region: a slope less steep than in the experiment (Figures S5 and S7). The χ^2^ between the calculated and experimental profiles effectively correlates with the deviation from the experimental scattering slope in the Guinier region (Figure 2), and therefore indicates the realism of the simulated protein size and expansion. Four MD models demonstrate χ^2^ ≤ 10, that is, an almost perfect overlap of the MD and experimental profiles (Figure 2F–I).

The influence of the water model on protein dimensions shows clearly on SAXS profiles. The standard TIP3P and TIP4P that underestimate COR15A WT dimensions (Figure 1) lead to SAXS profiles with χ^2^ ≥ 30. This is presumably due to an insufficient solvation, which the refined water models TIP4P/2005, TIP4P/2005s, a99SB-disp, and TIP4P-D overcome by an improved balancing of protein–solvent interactions.^18,38^ Interestingly, the CUFIX strategy to achieve the same by attenuating protein–protein interactions—thus allowing the continued use of the common and computationally cheaper 3-point water models—appears to, unfortunately, fail for COR15A WT.

#### 2.1.3 Secondary Structure

Experimental data indicate that COR15A WT is largely unstructured when fully hydrated. Secondary structure, if present, is limited to α-helix and strictly dependent on water availability.^27,28,39,40^ This is met by most MD models: if sampled, secondary structure is predominantly limited to helical elements. Exceptions are OPLS, ff99SB*-ILDN-Q, and ff99SB*-ILDN, which sample almost no helical, but 5–10% β-sheet structures (Figure S8).

In contrast to *R*_G_, which reached equilibrium after 120 ns in all MD models (Figure S3), α-helicity was increasing during the whole 200 ns simulation in CHARMM27, ff99SB-NMR, ff03ws, and both CUFIX force fields (Figure S9), indicating that helix formation, if occurring, is a rather slow process that cannot necessarily be captured within 200 ns without using enhanced sampling approaches.^41^ Validation-wise, this is not a problem for the six MD models that overestimated helical content (compared to α-helicity determined by NMR chemical shifts,^28^ Figure S9D,E,L,N,S,T), as they certainly exaggerate helicity—but it might lead to underestimation of helicity in other MD models. For this reason, we refrain from using α-helicity derived from short MD simulations as a validation parameter.

### 2.2 Extended Simulations of the Top-6 Models

In addition to the analysis of global structural properties, the verification of secondary structure and dynamics is critical for the evaluation of MD models. Based on the above-described validation via *R*_G_ and scattering profiles, we extended simulations of the six best-performing models (Figure 2) to a total of 10 × 1.2 μs each to further analyze their α-helix formation and protein dynamics. In addition to COR15A WT, we also included the point mutant G68A, which is not only more prone to acquire helical structure upon dehydration but, according to NMR^28^ and circular dichroism (CD) experiments (see ref 28 and Figure S10), has slightly higher transient helicity already in the fully hydrated state. We were specifically interested to see (i) if simulations were able to detect this rather small helicity difference; and, (ii) if it translates to measurable changes in protein dynamics in NMR relaxation experiments, (iii) are these also reproduced in simulations. In other words, could protein dynamics be correctly derived from contemporary all-atom MD simulations for highly folding-prone IDPs?

To ensure thorough equilibration, the first 200 ns of each simulation were designated as an extended equilibration period; the subsequent 1 μs was used for production analysis. During these extended simulations (Figure S11), no significant differences between COR15A WT and G68A were identifiable in *R*_G_ (Figures S12 and S13A).

#### 2.2.1 Distribution of α-Helicity

In the extended trajectories, both COR15A variants have a low but significant average α-helical content in all the six MD models, and β-sheet content in ff99SB*-ILDN-HMS-C_corr_^γ^+TIP4P/2005 and DES-amber (Figure S13B).

Figure S14 shows the progression of overall α-helicity during the extended simulations and compares this to the experimental α-helicity derived from backbone NMR chemical shifts.^28^ The experimental data underline the disordered character of both proteins, which, however, sample a detectable extent of transient helical structure. During the extended simulations, α-helicity appears equilibrated for all MD models except perhaps COR15A WT in ff99SB-disp; in ff99SBws and ff03ws, equilibrium is reached only after 400 ns for both proteins. These force fields, ff99SB-disp, ff03ws, and ff99SBws, also resulted in too high overall helicity (Figure S14A–C,G–I), similar to what we saw in the 200 ns simulations for the CHARMM27, ff99SB-NMR, and both CUFIX force fields (Figure S9D,E,L,N,S,T). Importantly, the slow process of helix formation has no major effect on *R*_G_ (Figure S12), supporting our usage of *R*_G_ from the 200 ns simulations (Figure 1) as the first validation parameter for our total set of 20 models. Three of the six tested MD models (DES-amber and both ff99SB*-ILDN-HMS-C_corr_^γ^ models) generated distributions of α-helicity significantly overlapping the experimental range (Figure S14D–F,J–L).

Figure 3 then shows the distribution of time-averaged α-helicity along the protein sequences in the simulations. It is compared against the distribution determined from experimentally observed backbone NMR chemical shifts,^28^ which indicate slightly higher helicity in G68A than WT between residues 55 and 70. The increased helicity is also reflected in the shapes of far-UV CD spectra (Figure S10), corroborating that the transition of chemical shifts is indeed due to changing helicity.

**Figure 3:**
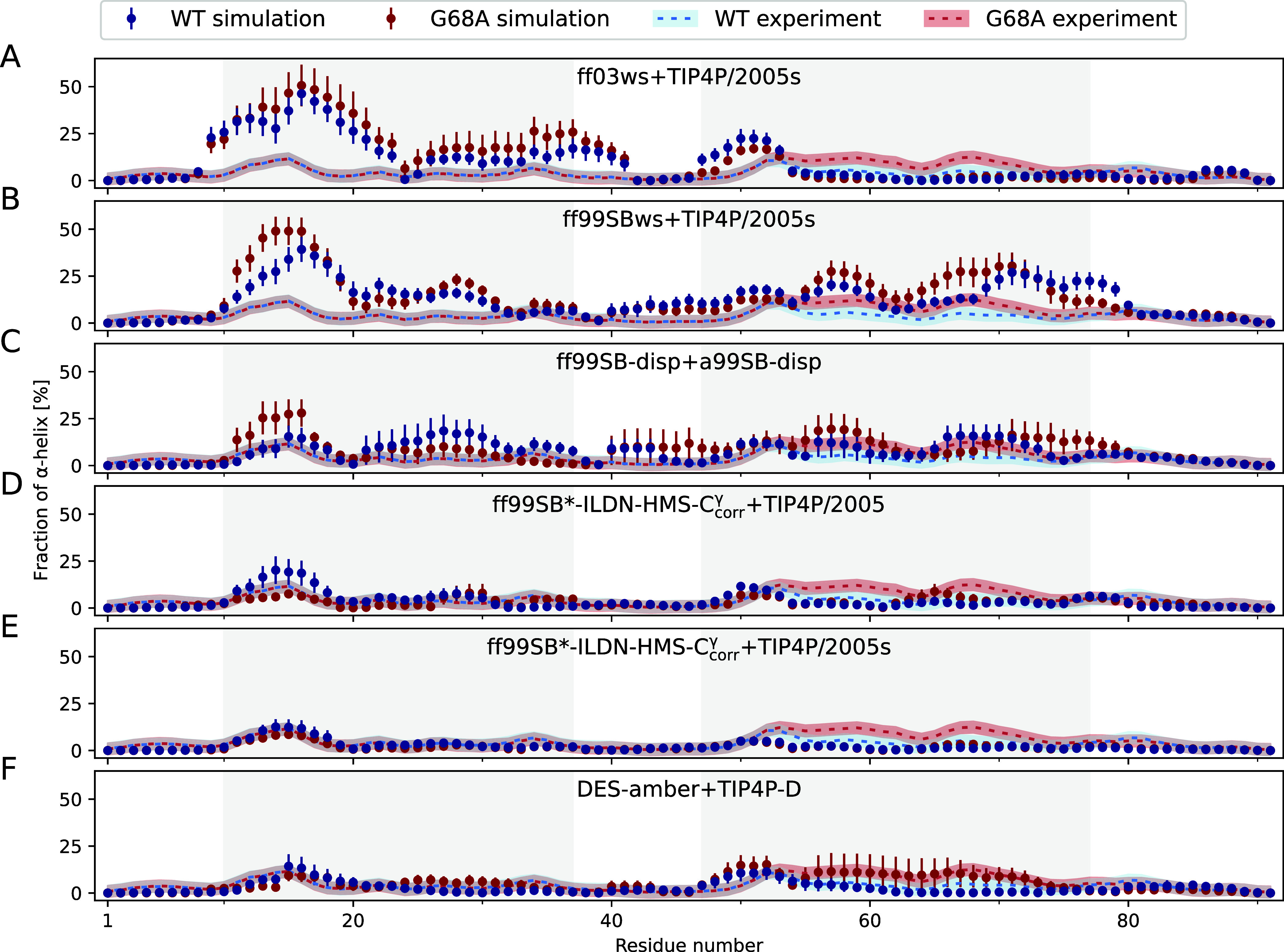
Fraction of α-helicity per residue determined as average over all extended simulation trajectories corresponding to the same MD model (circles) for COR15A WT (blue) and G68A (red) in comparison to the respective experimental data (dashed lines) (A–F). Errors for simulation show SEM, calculated from *n* = 10 independent repeats. Blue and red shaded areas indicate an experimental error estimate of 3%. Shaded regions in gray indicate experimentally identified protein segments prone to folding into α-helical structure in the presence of trifluoroethanol (TFE).^28^

The distinct oversampling of average helical structure seen in ff03ws, ff99SBws, and ff99SB-disp (Figure S14) can now be assigned to specific protein regions. For ff03ws, it results from the prediction of completely disordered C-terminal moieties and N-terminal parts with a rather high tendency to form α-helical structures, especially between residues 10 and 20 (Figure 3A). For ff99SBws, the N-terminal parts of both proteins are qualitatively similar, but they demonstrate a clear α-helix preference also for the C-terminal parts. These two regions of higher helicity, from residues 10–30 and 50–80, are separated by a region of lower helicity (Figure 3B), an arrangement hinting at the previously reported helix–loop–helix motif for the folded COR15A WT.^27,28,42^

Nevertheless, the other four MD models, which were specifically developed for MD studies of IDPs by adjusting the water–protein interaction terms and/or torsion parameters to precisely balance helix and coil states,^18,20,38,43^ achieve a better description of the experimental per-residue helicity distribution. Figure 3C illustrates a more uniformly distributed and lower tendency for α-helical structure in the central part (residues 10–80) for ff99SB-disp compared to the former two force fields. This results in helicity profiles that are in equally good agreement with the experimental data of both COR15A variants. However, it does not detect the small difference between COR15A WT and G68A. ff99SB*-ILDN-HMS-C_corr_^γ^ with TIP4P/2005 (Figure 3D), and especially TIP4P/2005s (Figure 3E), succeed in capturing the N-terminal short region of low but nonzero helicity (residues 10–20) with impressive accuracy, but not the longer one that both proteins apparently populate in their C-terminal halves. Strikingly, DES-amber (Figure 3F) not only yielded an excellent match with the experimental helicity profiles but was also able to precisely capture the small helicity difference between the two COR15A variants as measured in experiments.

#### 2.2.2 Chemical Shifts

Chemical shifts capture structural dynamics as they are sensitive to the local chemical environment and reflect both structural features and dynamic processes such as backbone conformation and hydrogen bonding. This renders them a robust basis for assessing the agreement between simulated IDP ensembles and experimental NMR data.^44,45^

Chemical shifts were predicted from MD simulation frames using SPARTA+^46^ (δ_sim_) and evaluated against NMR experiments^28^ (δ_exp_) for backbone amide ^15^N and ^1^H^N^ (Figures S15 and S16) and as △δ = δ_sim_ – δ_exp_, as shown in Figure 4.

**Figure 4:**
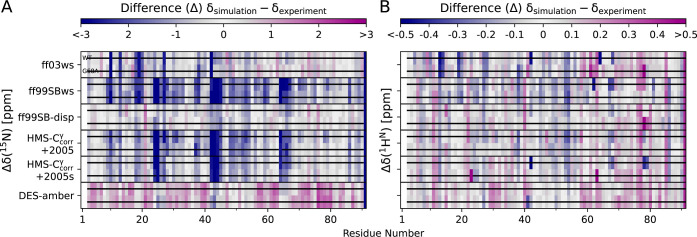
Comparison (simulation minus experiment) between per-residue chemical shifts δ of the backbone amide ^15^N and ^1^H^N^ predicted from MD simulation frames via SPARTA+^46^ and derived from experiments^28^ are represented as △δ­(^15^N) (A) and △δ­(^1^H^N^) (B) for the MD models ff03ws, ff99SBws, ff99SB-disp, ff99SB*-ILDN-HMS-C_corr_^γ^ (HMS-C_corr_^γ^), and DES-amber. For HMS-C_corr_^γ^, two water force fields TIP4P/2005 and TIP4P/2005s (referenced as 2005 and 2005s, respectively) are shown. In each individual force field panel, the upper row shows COR15A WT and the lower row G68A. A black dash indicates that the combined error (ε) from simulation (ε_sim_) and SPARTA+ prediction (ε_pred_) overlays the experimental chemical shifts; more precisely, that △δ ± ε with $\varepsilon =\sqrt{\varepsilon_{sim}^{2}+\varepsilon_{pred}^{2}}$ overlaps with zero. The simulation error is represented as SEM from *n* = 10 trajectories and prediction errors are 2.45 ppm for ^15^N and 0.49 ppm for ^1^H^N^;^46^ ε_sim_/ε_pred_ ≈ 0.1, such that ε is mainly dominated by ε_pred_.

While the experimental and simulation-predicted ^1^H^N^ chemical shifts to a large extent agree (Figure 4B), more pronounced differences between the MD systems are evident for ^15^N: A generally high consensus is observed for ff99SB-disp, similar to ff03ws, showing a few exceptional—but pronounced—outliers in the N-terminal region. DES-amber is the only MD system that slightly but systematically overestimates ^15^N chemical shifts. In contrast, ff99SBws and both ff99SB*-ILDN-HMS-C_corr_^γ^ systems considerably underestimate the ^15^N shifts in the central protein region (Figures 4A and S16A).

However, all MD systems indicate substantial overlap between simulation errors and experimental chemical shifts—as indicated by the black dashes—for ^15^N and ^1^H^N^ (Figure 4). This outcome is at least in part attributable to the inclusion of SPARTA+’s prediction uncertainty in the simulation error (Figure S15); with uncertainties^46^ (^15^N: 2.45 ppm and ^1^H^N^: 0.49 ppm) being approximately 10 times larger than corresponding SEMs across the trajectories.

In conclusion, all six MD systems generated COR15A WT and G68A ensembles, whose predicted chemical shifts agree with experimental NMR data within the uncertainty limits of SPARTA+.

#### 2.2.3 Capturing Protein Dynamics via Experiments

The dynamical properties of COR15A WT and G68A were experimentally investigated by measuring the longitudinal (T_1_) and transversal (T_2_) NMR spin relaxation times and the heteronuclear Overhauser effects (hetNOEs) for the assignable residues of ^15^N-COR15A WT and ^15^N-G68A in two magnetic fields with strengths of 600 and 850 MHz (Figure 5). T_1_ (spin-lattice relaxation) describes the return of nuclear spins from an excited state to the ground state. T_2_ (spin–spin relaxation) describes the loss of net magnetization through dephasing of excited nuclear spins. In folded parts of proteins, this dephasing is supported by the rather static but differing local magnetic fields of the different nuclei, and occurs thus quicker than in dynamic disordered regions, where the local fields fluctuate and thus are, on average, more similar. Thus, the T_2_ times of residues in ordered regions are lower compared to amino acids in disordered regions.^47^ For molecular motions faster than 10 ns, both T_1_ and T_2_ decrease when the molecular motion slows (Figure S17A); for slower motions, T_1_, but not T_2_, increases. Recently, a linear relation between effective correlation time and T_2_ has been reported.^47^ HetNOEs are (with the fields we used here) sensitive to protein dynamics in a time scale window of 100 ps–10 ns, with residues with smaller hetNOEs being involved in faster motions (Figure S17B).^48^

In the large central part of both proteins, T_1_ times are homogeneous throughout the sequence (Figure 5A), while T_2_ times are slightly higher between residues 35–50 than in the neighboring regions (Figure 5B). This difference is more distinct at 600 MHz than at 850 MHz. In both fields and proteins, higher values for T_1_ and T_2_ were observed in the N- and C-terminal tails as compared to the central parts. In all experiments, hetNOEs indicate a decrease only for the 10 C-terminal residues (Figure 5C).

**Figure 5:**
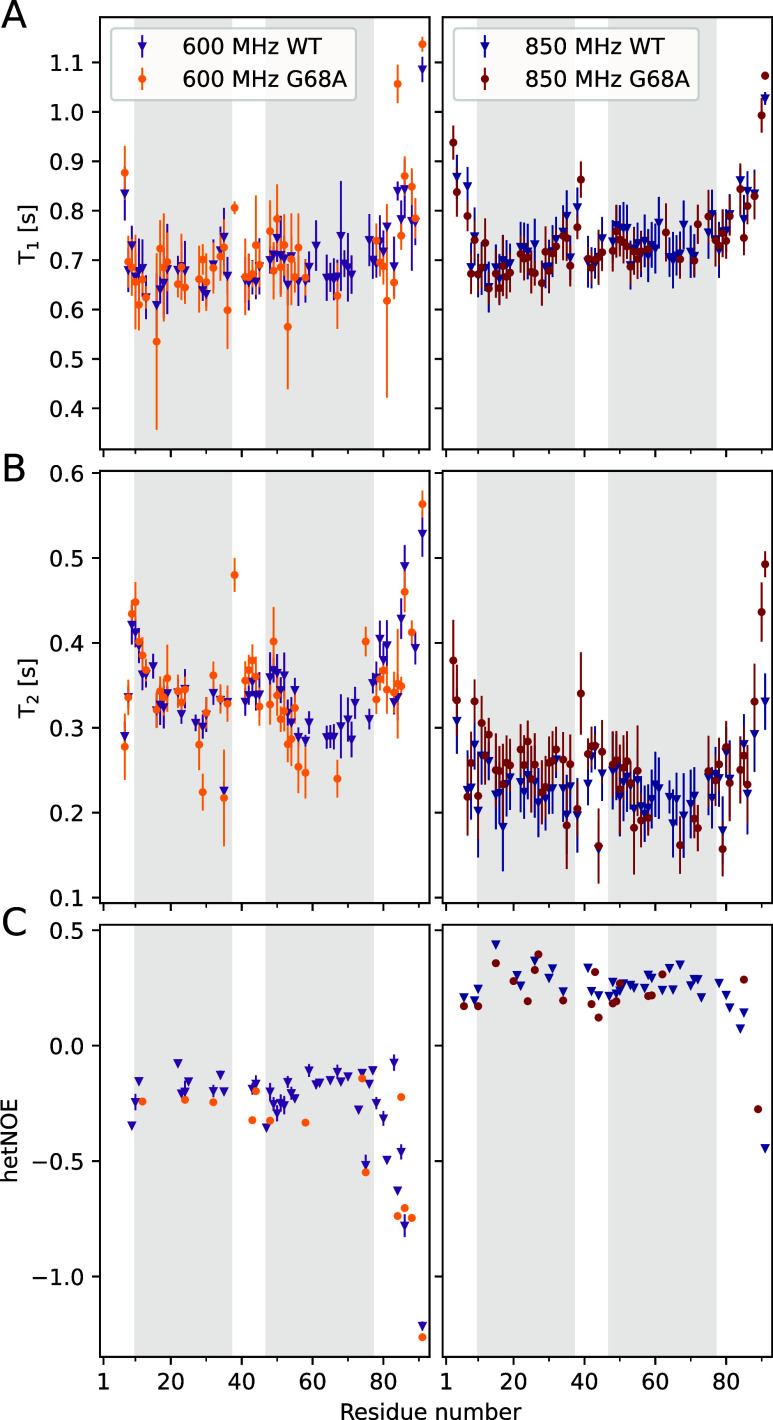
Comparison of experimentally determined NMR parameters T_1_ (A), T_2_ (B), and hetNOE (C) of ^15^N-COR15A WT and ^15^N-G68A residues measured at field strengths of 600 MHz (left) and 850 MHz (right). The error bars represent the experimental error, if applicable. Shaded regions in gray indicate experimentally identified protein segments prone to folding into α-helical structure in the presence of TFE.

Thus, the single-point substitution from glycine to alanine at position 68 does not affect the global main chain dynamics in the NMR experiments. This is surprising, as the substitution has an influence on the α-helicity, which is assigned to the C-terminal moiety.^28^ Unfortunately, the lack of experimental spin relaxation data of G68A in the vicinity of the mutation site hinders direct comparison of local dynamics from experimental data.

#### 2.2.4 Evaluation of Simulated Protein Dynamics Compared to Experimental Data

MD simulations yield information about the correlation of molecular motions over time. In particular, the rotational correlation of an N–H bond during simulation can be used to reconstruct the spectral density function (eq 9), which then provides the T_1_, T_2_, and hetNOE via the Redfield equations.^49,50^ In this context, it is important to note that different field strengths sample the spectral density function differently: the T_1_, T_2_, and hetNOE are sensitive to different molecular motions at 600 MHz than at 850 MHz.

Figures S18 and S19 compare the per-residue T_1_, T_2_, and hetNOE computed from the extended simulations against the experimental values; the corresponding differences △T_1_, △T_2_, and △hetNOE are shown in Figure 6. As T_1_ and T_2_ are typically closer to experimental values at 850 MHz than at 600 MHz, and differences between COR15A WT and G68A are minor, let us focus first on the 850 MHz COR15A WT comparison.

**Figure 6:**
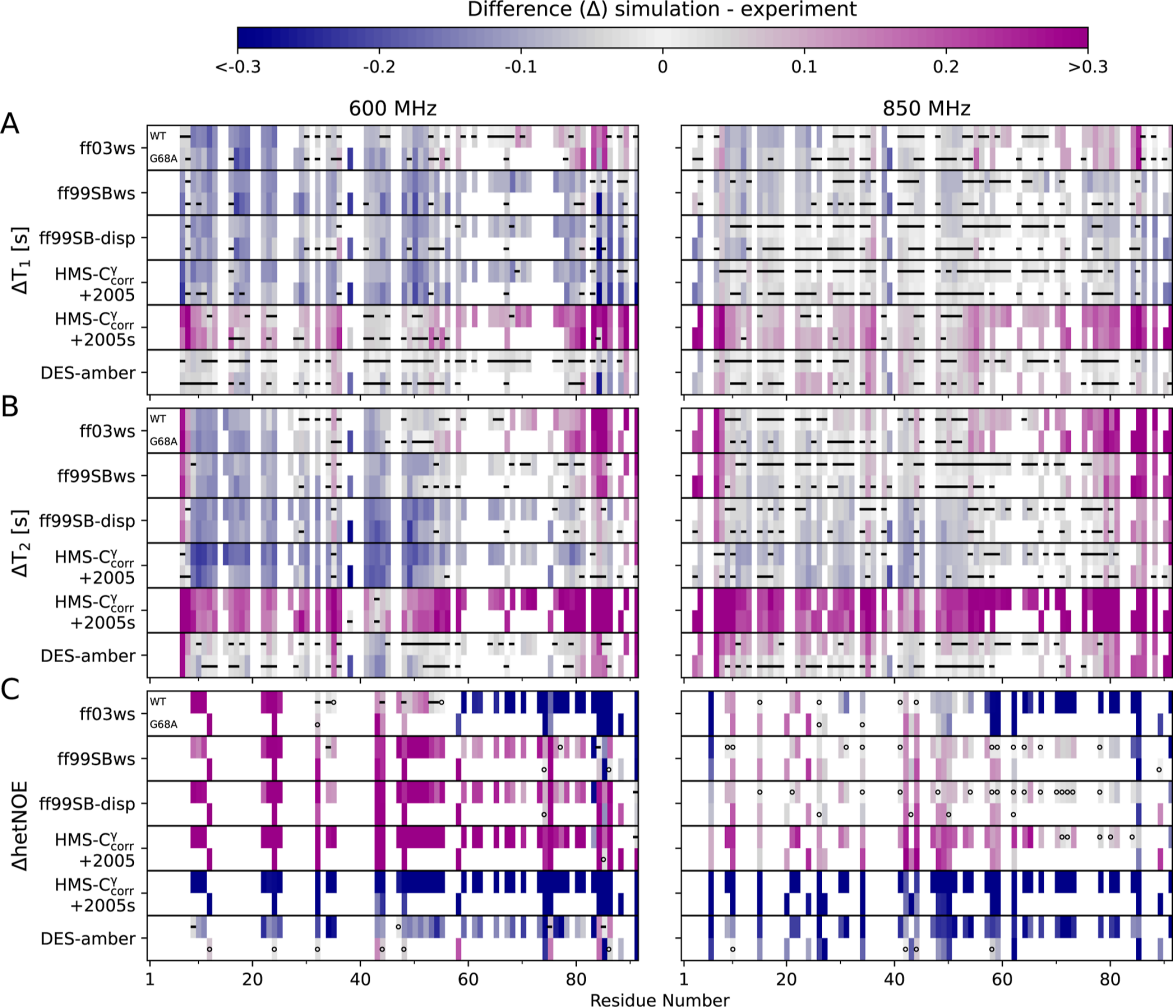
Comparison (simulation minus experiment) between per-residue NMR relaxation parameters calculated from MD simulations and derived from experiments is represented as △T_1_ (A), △T_2_ (B), and △hetNOE (C) for the MD models ff03ws, ff99SBws, ff99SB-disp, ff99SB*-ILDN-HMS-C_corr_^γ^ (HMS-C_corr_^γ^), and DES-amber. For HMS-C_corr_^γ^, two water force fields, TIP4P/2005 and TIP4P/2005s (referenced as 2005 and 2005s, respectively), are shown. In each individual force field panel, the upper row shows COR15A WT and the lower row G68A in two field strengths of 600 (left) and 850 MHz (right). A black dash indicates that the simulation (ε_sim_) data overlay the experimental (ε_exp_) errors; more specifically, △ ± ε, with $\varepsilon =\sqrt{\varepsilon_{\exp}^{2}+\varepsilon_{sim}^{2}}$ , overlaps with zero. When an experimental error could not be determined, an empty circle demonstrates an overlay of the experimental value with the simulation error bars.

We base this comparison mainly on △T_1_, △T_2_, and △hetNOE (Figure 6), but also discuss two features computed from simulations: the rotational time scale distributions for all backbone N–H bonds (Figures 7 and S20) and the directional correlation of vectors between two consecutive Cα carbons across the whole sequence (Figure 8). The latter carries information on the rigidity of the conformational ensemble, the former on the time scale distribution of typical molecular motions. Our simulations mostly sampled rotational time scales in the 0.1–10 ns range, as previously reported for IDPs,^51^ but differences in time scale distributions are clear between the MD models (Figure S20).

**Figure 7:**
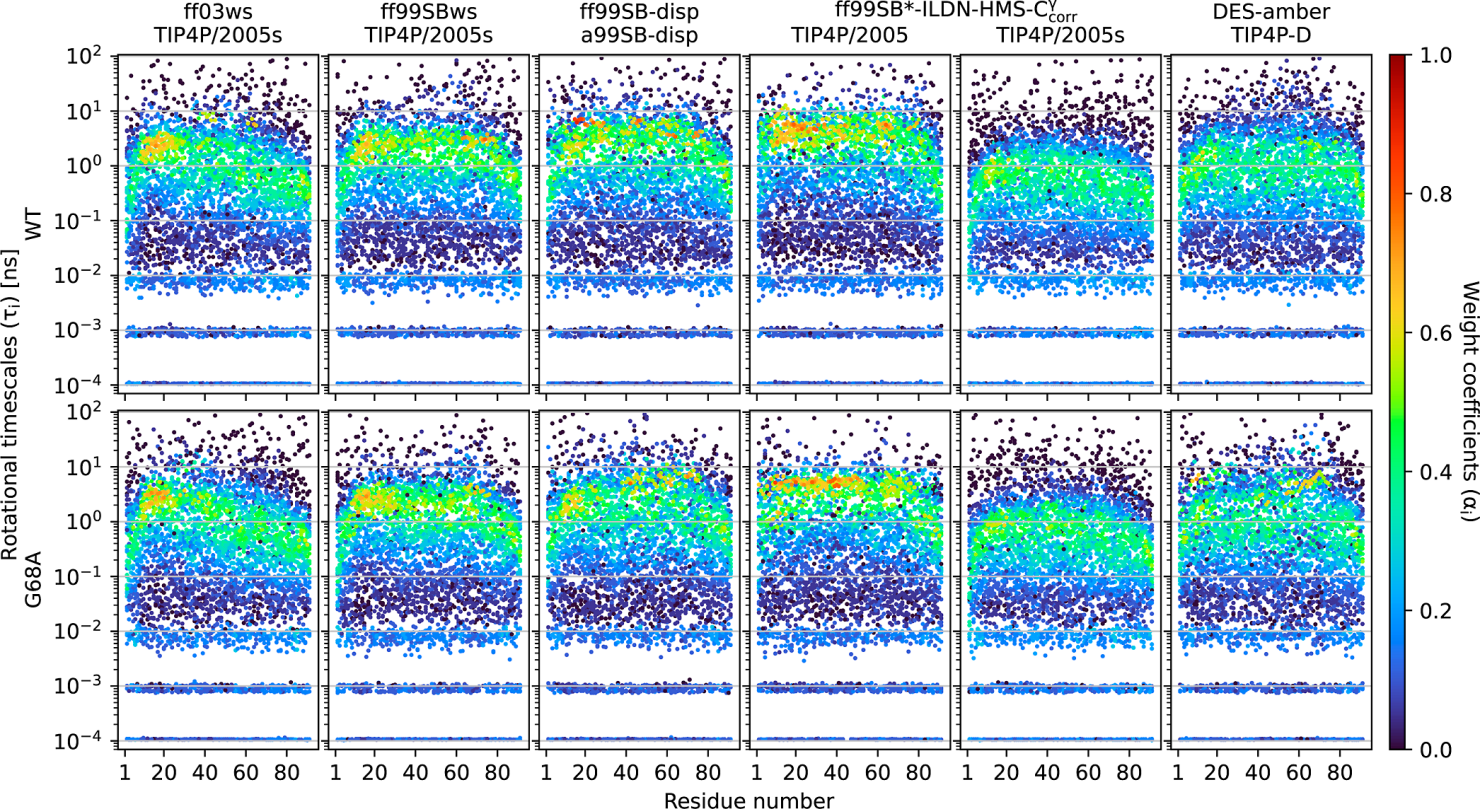
Rotational time scales (τ_*i*_) calculated according to eq 11 from MD simulations of COR15A WT and G68A with related weight coefficient α_*i*_ > 0 are shown per residue. The corresponding weight coefficient α_*i*_ of every data point is depicted by a color gradient. Demonstrated are the individual results of 10 simulations with the MD models ff03ws, ff99SBws, ff99SB-disp, ff99SB*-ILDN-HMS-C_corr_^γ^ in combination with the water models TIP4P/2005 and TIP4P/2005s, and DES-amber.

**Figure 8:**
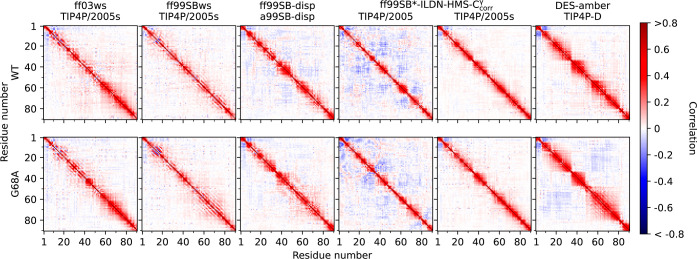
Directional correlation maps of vectors between consecutive Cα carbons during extended MD simulations of COR15A WT (upper row) and G68A (lower row) using the force fields ff03ws, ff99SBws, ff99SB-disp, ff99SB*-ILDN-HMS-C_corr_^γ^ in combination with the water models TIP4P/2005 and TIP4P/2005s, and DES-amber. Values on the abscissa and ordinate are residue numbers. Correlations were calculated from 10 simulation replicates for each protein.

Of the six selected MD models, ff99SB*-ILDN-HMS-C_corr_^γ^ + TIP4P/2005s shows the most deviating △T_1_, △T_2_, and △hetNOE at 850 MHz: It slightly overestimates T_1_, drastically overestimates T_2_, and underestimates hetNOEs (Figure 6). This indicates excessive flexibility manifested by rotational time scales faster than in the other five MD models (Figure S20), weak orientational backbone correlations (Figure 8), and a slight under-representation of transient helicity (Figures 3 and S14). Notably, ff99SB*-ILDN-HMS-C_corr_^γ^ was originally developed in combination with TIP4P/2005, but aligned better with experimental *R*_G_ data when paired with TIP4P/2005s (Figure S12); unfortunately, the switch to TIP4P/2005s appears to disrupt dynamics.

Of the other five MD models, the C-terminus in ff03ws shows a similar rotational time scale fingerprint as ff99SB*-ILDN-HMS-C_corr_^γ^ + TIP4P/2005s at 850 MHz (Figure 6), indicative of sampling shorter-than-experimental rotational correlation time scales (Figures S19 and S20). Interestingly, these fast dynamics are accompanied by a substantial rigidity (Figure 8), which is, however, not due to helicity (Figures 3 and S14).

Simulations with the other four MD models show a relatively good fit with the experimental T_1_ and T_2_ data collected at 850 MHz (Figure 6). The sampled rotational time scales increase in the order DES-amber < ff99SBws ≤ ff99SB-disp < ff99SB*-ILDN-HMS-C_corr_^γ^ + TIP4P/2005 (Figures 7 and S20). DES-amber seems to capture experimental T_1_ times slightly better than the other three models in the C-terminal half (Figure 6). Experimental hetNOEs are not available for all residues; however, the available hetNOEs at 850 MHz are best captured by ff99SB-disp and ff99SBws, while ff99SB*-ILDN-HMS-C_corr_^γ^ + TIP4P/2005 slightly overestimates and DES-amber underestimates them (Figure 6).

The good agreement of simulated relaxation parameters with the experimental 850 MHz NMR data in five of the six MD models (with ff99SB*-ILDN-HMS-C_corr_^γ^ + TIP4P/2005s as the exception) (Figure 6) indicates accurate sampling of the rotational time scales sensitive to the 850-MHz field. Notably, even though the overall performance of ff99SB*-ILDN-HMS-C_corr_^γ^ + TIP4P/2005s is inadequate, it does partially agree with the experiments at 600 MHz, that is, it samples better the rotational time scales sensitive for the 600 than the 850 MHz field strength. DES-amber was the only model that yielded good results for both magnetic fields (except for the hetNOEs at 850 MHz).

Overall, while DES-amber performs as well as the best MD models for T_1_ and T_2_ at 850 MHz, it is the only model that captures COR15A dynamics also at 600 MHz. Interestingly, DES-amber also detects slight structural differences between COR15A WT and G68A in the C-terminal half of the proteins (Figures 3 and 8).

### 2.3 Interpretation of COR15A Dynamics by Integrating Experiment and Simulation

Let us then interpret experimental relaxation data through the best performing MD model, DES-amber, to derive information on the complex dynamics of COR15A at subnanometer–subnanosecond resolution, which are not readily apparent from experimental data.^51^ Our analysis of rotational time scale distributions (Figures 7 and S20) indicates that COR15A samples a broad range of rotational time scales, as reported for IDPs previously.^10,51–53^ Both termini sample faster time scales than the midchain, agreeing with a sterically less constrained movement.

Apart from neighboring residues, directional correlation is present but rather weak (Figure 8), suggesting a low but non-negligible degree of long-range interaction in terms of hydrophobic or electrostatics within the conformational ensembles at neutral charge conditions. The slightly higher propensity of the C-terminal half of G68A for transient helicity (Figure 3) compared to COR15A WT is reflected in higher directional correlation (Figure 8). While a comparison of local dynamics of COR15A WT and G68A is not possible from experimental data due to the low data density of G68A in the vicinity of the mutation site (Figure 5), we performed such a comparison based on relaxation data simulated with DES-amber, encouraged by its good performance (Figure S21). Indeed, the simulated T_1_ times are slightly higher in G68A compared to COR15A WT at 850 MHz, but not at 600 MHz in the proximity of the mutation site; T_2_ times and hetNOEs are comparable in both field strengths. This suggests that the mutation locally affects molecular motions with time scales slower than about 10 ns (see Figure S17).

## 3 Conclusions

Our major aim was to explore the performance of MD models developed for the study of disordered proteins in the context of highly folding-prone IDPs. To this end, we simulated the folding-prone COR15A in 20 different MD models and found that systems using water force fields specifically developed for disordered proteins can accurately capture its dimensions. Interestingly, improving solvation via adapted water models seems to work better than attenuating protein–protein interactions (Figures 1 and 2).

IDPs are not only characterized by their conformational ensembles but also, to a large extent, by the dynamics within these ensembles. Our work pioneers the use of ensemble dynamics in comprehensive force field comparison studies on IDPs. Our NMR data set of COR15A dynamics will pave the way for the development of future force fields for IDPs, especially those at the verge of folding.

Ensemble dynamics were assessed by direct computation of the relaxation parameters T_1_, T_2_, and hetNOE from all-atom MD simulation trajectories, as previously done for ordered states and proteins containing IDRs;^13,51,54^ our study confirmed the suitability of this approach also for fully unstructured proteins. Importantly, we found that for COR15A, spin relaxation data can distinguish between simulations that give equally good agreement with SAXS data, suggesting that evaluation against NMR relaxation is more sensitive than against SAXS. We find satisfactory agreement of simulation and experiment with respect to protein dynamics for the DES-amber + TIP4P-D model (Figure 9).

**Figure 9:**
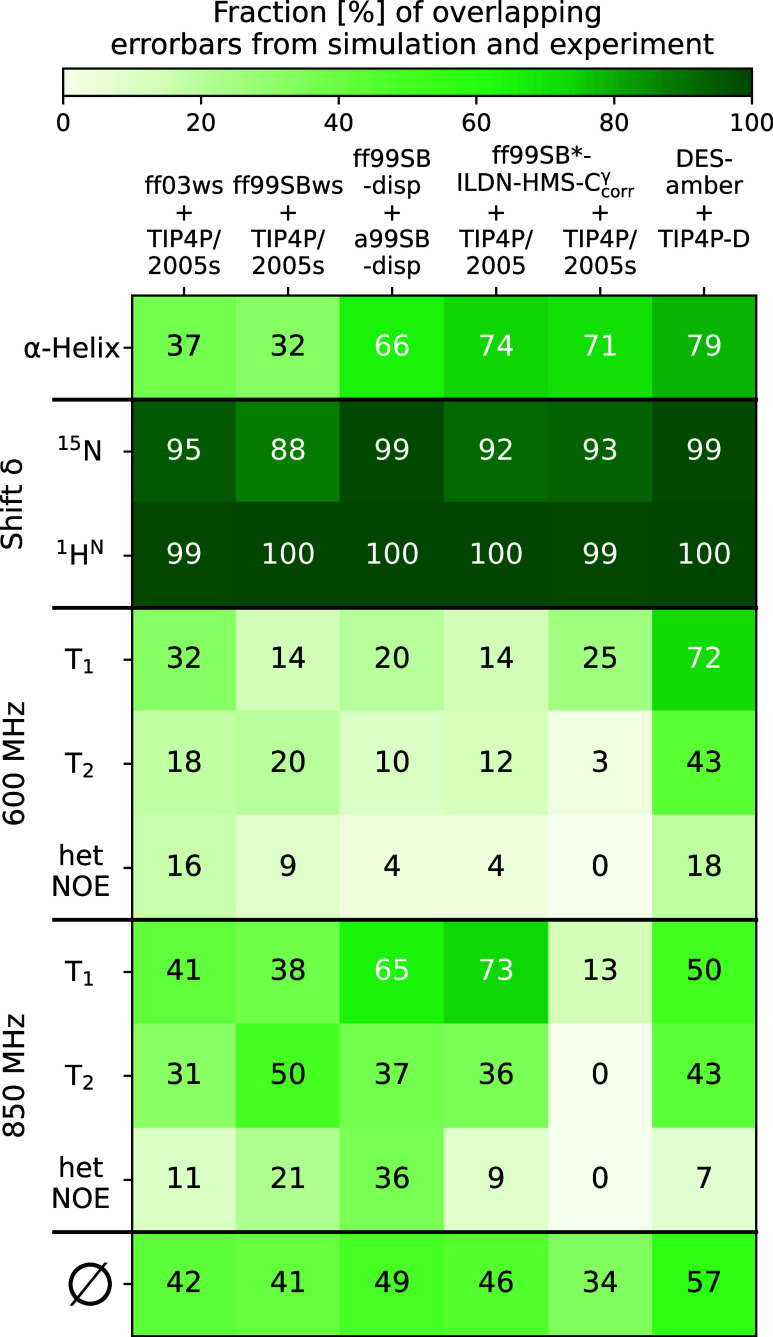
Summary of agreement between extended MD simulations and experimental NMR data expressed as the fraction of overlapping error bars from simulation and experiment, averaged over COR15A WT and G68A. The last row presents the overall agreement, calculated as the average (Ø) across all individual evaluations for each MD system.

It is worth noting the major differences the water force field can cause in dynamics, as exemplified by ff99SB*-ILDN-HMS-C_corr_^γ^: With the TIP4P/2005 water model, it is quite close to experimental data, but TIP4P/2005s results in too fast dynamics (Figure 7). Notably, with regard to overall protein dimensions derived from SAXS, TIP4P/2005 samples overly compact ensembles, but TIP4P/2005s matches the experimental data with high accuracy (Figures 1 and 2). This ambivalence underscores the necessity to validate MD outcomes against a comprehensive set of structural and dynamic experimental data.

Although several MD models developed for IDPs described the conformational ensembles of our folding-prone IDPs rather well (Figure 2), only DES-amber achieved an overall satisfactory overlap with both the structural and dynamical experimental data. Interestingly, it also detected minor structural differences between protein variants, such as the small increase of α-helicity in the C-terminus caused by the G-to-A mutation suggested by earlier NMR and CD experiments.^28^ This helicity difference between the two variants does indeed translate to modulation of dynamics, a finding that could not be deduced from experiments due to a lack of data density (Figure 5), but was detectable from simulations (Figure S21). Also, some differences in ensembles between the variants can be captured via the directional correlation along the protein backbone (Figure 8), an observable exclusively accessible by simulation.

Simulation-predicted backbone amide chemical shifts showed strong agreement with experimental NMR data, although this apparent consistency likely stems from the broad prediction error of SPARTA+ (Figures 4 and S15). Consistent with other studies,^45,55^ this suggests that relying solely on chemical shifts is not sufficient for force field evaluation and therefore a broader range of parameters should be considered.

An interesting next step will be to evaluate the accuracy of DES-amber in modeling dehydration-induced COR15A folding and rehydration-induced unfolding.

## 4 Experimental Section

### 4.1 MD Simulations

#### 4.1.1 Initial Structures

The mature amino acid sequence (91 amino acids, see Figure S22) of COR15A without the N-terminal chloroplast transit peptide was obtained from the TAIR database (Protein ID: AT2G42540.2).^56^ For the G68A point mutant, glycine (G) at position 68 was substituted by alanine (A).

To generate ensembles of 10 initial protein structures that reflect the disordered nature of COR15A WT (Figure S23) or G68A (Figure S24),^16,57,58^ straight-chain conformations were created with protein backbone dihedral angles of 180° by PyMol’s fab function^59^ (ss = 4) and centered in a rectangular simulation box generated by Gromacs editconf tool^60^ with a minimum protein–edge distance of 15 Å. For net charge neutrality, six sodium ions were added by Gromacs genion.^60^ Ten 150 ns MD simulations with independent initial velocities were run using Gromacs 2018.7 in TIP4P^61^ water in combination with the OPLS-AA force field,^37,62,63^ the Bussi–Donadio–Parrinello^64^ thermostat (300 K, τ_*T*_ = 0.1 ps) and the Parrinello–Rahman barostat^65^ (1 bar, τ_*P*_ = 2.0 ps), similar to previous MD studies of COR15A WT^27,42^ (for equilibration, see Figure S25).

From the 10 corresponding trajectories, individual protein structures were sampled in steps of 3 ns, starting at 80 ns when the mass-weighted RMSD of protein backbone atoms stabilized, yielding 250 conformations for both the COR15A WT and G68A mutant. The heterogeneity of the resulting initial structural ensemble, of special importance for IDPs, was confirmed by analyzing the distribution of *R*_G_ (Figure S26). In addition, these two sets were hierarchically clustered using MaxCluster^66^ with single-linkage and the TM-score^67^ as distance parameter (Figure S27). The centromeres of the 10 resulting clusters were selected as starting structures for all further simulations. No statistical weighting of conformations from different clusters was performed. Instead, we followed the common practice of unweighted (or random) sampling,^68,69^ but verified that the initial ensemble conformations are heterogeneous, independent, and not biased toward extreme states. For further details, see Section S1.

#### 4.1.2 Equilibration and Production

All MD simulations were run on Gromacs 2018.7/.8 or 2022.3/.4 ^60^ in fully periodic dodecahedral boxes with an initial protein–edge distance of 20 Å. Net charge neutrality was again guaranteed by adding six sodium ions (genion algorithm^60^). After energy minimization by steepest descent within the OPLS-AA force field, models were solvent-equilibrated, first 1 ns in NVT, then 1 ns in NPT, with protein heavy atoms position-restrained (1000 kJ mol^–1^ nm^–2^).

The in-NVT-and-NPT-equilibrated models were used as input for Gromacs pdb2gmx to change the force field and the explicit water model to sTIP3P,^61^ mTIP3P,^17^ TIP4P,^61^ TIP4P/2005,^70^ TIP4P/2005s,^18^ TIP4P-D,^38^ or a99SB-disp.^20^ If necessary, virtual sites (transition from TIP4P to TIP3P) were removed. All created MD models and parameters for the productions are given in Table 1.

The 10 in-NVT-and-NPT-equilibrated MD models were then used for 200 ns (see Section 2.1 and Table 1) and further 1 μs (see Section 2.2) production runs conducted at 300 K and 1 bar with a time step of 2 fs. The Coulomb interactions were treated with particle-mesh Ewald^80^ (10 Å real-space cutoff), van der Waals interactions with a 10 Å cutoff combined with a force-switching function of 10 Å. The first 200 ns of 1.2 μs simulations were not considered for dynamic and structural analyses.

**Table 1:** Tested MD Models with Corresponding Barostat and Thermostat Parameters^a^

#	force field	water model	thermostat	τ_T_ (ps)	barostat	τ_p_ (ps)
1	OPLS-AA^37,62^	TIP4P^61^	BDP	0.1	PR	2.0
2	CHARMM22*^71^	mTIP3P^17^	NH	1.0	PR	5.0
3	CHARMM22*^71^	TIP4P^61^	NH	1.0	PR	5.0
4	CHARMM27 ^72,73^	mTIP3P^17^	NH	1.0	PR	5.0
5	CHARMM27 ^72,73^	TIP4P^61^	NH	1.0	PR	5.0
6	CHARMM36m^74^	mTIP3P^17^	NH	1.0	PR	5.0
7	CHARMM36m^74^	TIP4P^61^	NH	1.0	PR	5.0
8	ff99SB*-ILDN^75^	TIP4P^61^	BDP	0.1	PR	2.0
9	ff99SB*-ILDN-Q^76^	TIP4P^61^	BDP	0.1	PR	2.0
10	ff99SB*-ILDN-HMS-C_corr_^γ^ ^21,22^	TIP4P/2005 ^70^	BDP	0.1	PR	2.0
11	ff99SB*-ILDN-HMS-C_corr_^γ^ ^21,22^	TIP4P/2005s^18^	BDP	0.1	PR	2.0
12	ff99SB-NMR^77^	TIP4P^61^	BDP	0.1	PR	2.0
13	ff03ws^18^	TIP4P^61^	BDP	0.1	PR	2.0
14	ff03ws^18^	TIP4P/2005s^18^	BDP	0.1	PR	2.0
15	ff99SBws^18^	TIP4P^61^	BDP	0.1	PR	2.0
16	ff99SBws^18^	TIP4P/2005s^18^	BDP	0.1	PR	2.0
17	ff99SB-disp^20^	a99SB-disp^20^	BDP	0.1	PR	2.0
18	DES-amber^43^	TIP4P-D^38^	BDP	0.1	PR	2.0
19	ff99SB*ILDN-phi-CUFIX^23^	sTIP3P^61^	BDP	0.1	PR	2.0
20	ff14SB-OL15-CUFIX^23^	sTIP3P^61^	BDP	0.1	PR	2.0

a For further force field details, see Section 4.1.3. Each simulation with a length of 200 ns (first 120 ns equilibration, last 80 ns production) out of 10 repeats with independent initial velocities was run with 38,788 water molecules and 6 sodium ions. Trajectories are openly available at https://doi.org/10.17617/3.4I538Z. BDP: Bussi–Donadio–Parrinello,^64^ NH: Nosé–Hoover,^78,79^ PR: Parrinello–Rahman.^65^

Temperature *T* was controlled by the Nosé–Hoover^78,79^ (τ_*T*_ = 1.0 ps) thermostat for the CHARMM models and by Bussi–Donadio–Parrinello^64^ (τ_*T*_ = 0.1 ps) for others (Table 1), and pressure *P* by the (isotropically applied) Parrinello–Rahman barostat^65^ with τ_*P*_ = 5.0 ps for CHARMM and τ_*P*_ = 2.0 ps for all others (Table 1). Constraints of covalent bonds to their equilibrium lengths were applied by the (fourth-order single-iteration) parallel linear constraint solver­(P-LINCS)^81^ in proteins (H-containing bonds in CHARMM models, all bonds in others) and to their equilibrium lengths and angles by SETTLE^82^ in water.

All production simulations were performed on the HPC extension clusters of the Max Planck Computing and Data Facility.

The simulation setup in terms of the selected water and protein force fields of the MD models used in this study are described in the following sections and are listed in Table 1.

#### 4.1.3 On Force Fields

The following brief overview of the used force fields contextualizes, for the interested reader, a comparative analysis of them.

The CHARMM 22*^71^ force field is a modification of CHARMM 22 ^17^ with backbone torsion potentials improved for the helix–coil balance and suppressing overestimation of α-helical structure. In previous studies, including simulations on IDP ensembles, CHARMM 22* was positively evaluated against experimental NMR and SAXS data.^16,71^

A different expansion of CHARMM 22 is CHARMM 27,^72^ also known as CHARMM22/CMAP, where a CMAP (grid-based energy correction map) backbone correction was developed based on quantum mechanical (QM) calculations.^71,72^

Our third force field from the CHARMM family is CHARMM36m,^74^ which was specifically developed for both folded and intrinsically disordered proteins.^74^ It is based on CHARMM36,^83^ descendant of CHARMM22. CHARMM36m was positively validated by simulations of four different IDPs and the results were in good agreement with NMR and SAXS data.^74^

From the Amber force field family, we tested ff99SB*-ILDN,^75^ one of the first Amber models able to simulate the behavior of disordered proteins.^75^ Compared to its ancestor ff99SB,^84^ it has QM-optimized side-chain torsion potentials of the residues I, L, D, and N.^75^

For ff99SB*-ILDN-Q,^76^ the ILDN-variant was further updated by refitting side-chain torsion angles for the residue D and the partial charges for the charged residues (D, E, K, and R), resulting in improved α-helical propensity.^76^

Another development based on ff99SB*-ILDN^75^ is ff99SB*-ILDN-HMS-C_corr_^γ^,^21,22^ in which the rotations of side-chain methyl groups—specifically those accessible through NMR spin relaxation—were optimized against the corresponding relaxation data. This refinement was realized in two steps: First, Hoffmann et al. reparameterized the rotational barriers in AMBER ff99SB*-ILDN to achieve accurate methyl dynamics using CCSD­(T) coupled cluster calculations as a reference.^22^ Then Kümmerer et al. further optimized these parameters by comparing NMR relaxation rates from MD simulations with deuterium relaxation measurements of T4 lysozyme, demonstrating that small modifications to C^γ^ methyl groups can improve agreement with experiments.^21^

The original ff99SB was also modified by reweighing parameters based on a comparison between chemical shifts measured by NMR and determined from MD, yielding ff99SB-NMR.^77^

A different approach, used for improving ff03w^85^ and ff99SB*-ILDN-Q simulating IDPs, was the modification of short-range protein–water interactions (by changing the corresponding Lennard-Jones parameters), and adjustment of the solvent structure, yielding the water model TIP4P/2005s.^18^ The results were in good agreement with SAXS and FRET data; moreover, free energies of solvation were improved.^18^ The resulting force fields were named ff03ws^18^ and ff99SBws.^18^

The ff99SB-disp^20^ force field was developed for both folded and disordered proteins. Good agreement with experimental data for the disordered state, while maintaining state-of-the-art accuracy for folded conformations, was achieved by changes in torsion parameters and slight modifications in the protein–water van der Waals terms compared to ff99SB-ILDN.^38^ Additionally, the TIP4P-D^38^ water model was adjusted for simulations with the ff99SB-disp protein force field, yielding the a99SB-disp water model.^20^

The DES-amber force field was found suitable for folded and disordered proteins^43^ and is, in addition, able to describe protein–protein and protein–DNA interactions. Its development started from a variant of the Amber FF^86^ by recalculating backbone partial charges and Lennard-Jones parameters based on a large set of QM data; backbone torsions were modified to better match residue-specific Ramachandran maps.^87^

In the above improvements, capturing the dimensions of IDPs mostly went hand in hand with enhancing the IDP solvation by strengthening the protein–water interaction. The CUFIX force field strategy, in contrast, weakens the Lennard-Jones potentials of hydrophobic protein–protein contacts to match the osmotic pressures of amino acids. This allows for the confirmed use of well-established and computationally cheaper water models like TIP3P.^23^ The CUFIX rescaling method is available for the force fields ff99SB*ILDN-phi^75,88^ and ff14SB-OL15,^89^ resulting in ff99SB*ILDN-phi-CUFIX and ff14SB-OL15-CUFIX, respectively.^23^

Finally, the OPLS-AA force field^37,62^ was successfully used in previous MD studies to describe the unfolding of COR15A.^27,42^ It is modified based on the Amber all-atom force field^90^ for specifically describing the interaction between proteins and diverse types of solvents,^37,62^ but was not specifically parametrized for the simulation of IDPs.

### 4.2 Analysis of Trajectories

#### 4.2.1 Simulation Monitoring

For each MD simulation, the mass-weighted RMSD of protein backbone atoms was computed by the Gromacs rms tool,^60^ using the structure at the beginning of each trajectory as the reference.

The minimum distance from the protein to its closest periodic image was analyzed over time via the mindist procedure with parameter -pi implemented in Gromacs.^60^ The fraction of trajectory frames with the minimum distance <10 Å was below 5% for all MD systems, except for the 1.2-μs DES-amber simulations (8%), where it was confirmed to have no significant effect on the NMR relaxation; see Figure S28.

#### 4.2.2 Radius of Gyration *R*_G_

*R*_G_ represents the mass-weighted average distance of atoms to the center of mass of a molecule and is a measure of protein compactness.^91,92^ *R*_G_ in equilibrium (from 120 ns for initial analysis of 20 models and from 200 ns for the extended analysis of 6 models) were computed using the Gromacs gyrate tool; they were compared against typical *R*_G_-values of folded and denatured globular proteins with the molecular mass identical to the one of COR15A, as calculated from the scaling law^30,31,93^ (eq 1)­

1 $$R_{G}=R_{G0}\cdot N^{\nu }$$

with *N* = 91 representing the number of amino acids. For denatured globular proteins,^31^ the scaling factor *R*_G0_ = 2 and the scaling exponent ν = 0.598 ± 0.028; for folded proteins^30^ *R*_G0_ = 3 and ν = 1/3. The error of the folded *R*_G_ approximation is estimated from the distribution width at *N* = 91 from Figure 1a of Dima and Thirumalai,^30^ with an upper error of 5.5 and a lower error of 2 Å.

#### 4.2.3 α-Helicity

The fraction of α-helicity per residue in equilibrium (from 120 ns onward for initial analysis of 20 models and from 200 ns onward for the extended analysis of 6 models) was computed using the Gromacs do_dssp tool,^60^ which implements the Define Secondary Structure of Proteins (DSSP) algorithm determining the secondary structure of the protein based on the atom coordinates.^94,95^

#### 4.2.4 SAXS Profiles

To calculate the average SAXS profile from the conformational ensemble provided by an MD model in equilibrium (120–200 ns or 200 ns–1.2 μs, respectively), single simulation frames were first extracted as pdb files by Gromacs trjconv. The individual scattering curves of each such single structure were determined by crysol^96^ (version 3.1.3), setting the number of spherical harmonics to 50 (-lm 50), the order of the Fibonacci grid to 18 (-fb 18), and the number of data points for the output profile to 76 (-ns 76). The resulting profiles were averaged to yield the SAXS scattering profile *I*_comp_ of the ensemble and its SEM ε_comp_ (*n* = 10).

Accurate calculation of the SAXS profile from MD conformations must consider the scattering effects of the water shell; these primarily influence the Guinier region (for unfolded proteins: *qR*_G_ < 1.1 ^33^), and thus the *R*_G_.^34^ As in CRYSOL, which we used here, the water shell is accounted for by an envelope of electron density,^96^ the calculated scattering profiles should match the experimental one.

Computed SAXS profiles were then fitted (*I*_fit_) to experimental ones via the lmfit python package^97^ and the Levenberg–Marquardt^98,99^ approach using eq 2

2 $$I_{fit}=m\cdot I_{comp}+n$$

where the amplitude scaling factor *m* is correlated with the protein concentration in experiments.^100^ The offset correction *n* is used, as the experimental buffer subtraction is prone to errors at higher values of the scattering vector *q*.^100,101^

The fit was weighted (*w*) against the experimental error ε_exp_ and ε_comp_ (eq 3)­

3 $$w\left(q\right)=\frac{1}{\sqrt{\varepsilon_{comp}{\left(q\right)}^{2}+\varepsilon_{\exp}{\left(q\right)}^{2}}}$$

The fit quality was evaluated by calculating the unitless χ^2^ value of the experimental and fitted scattering profiles (eq 4)­

4 $$\chi^{2}=\sum_{q}{\left(\frac{I_{fit}\left(q\right)-I_{\exp}\left(q\right)}{\sqrt{\varepsilon_{comp}{\left(q\right)}^{2}+\varepsilon_{\exp}{\left(q\right)}^{2}}}\right)}^{2}$$

and the *q*-specific residuals (eq 5)­

5 $$Res\left(q\right)=I_{\exp}\left(q\right)-I_{fit}\left(q\right)$$

To calculate the fit error ε_fit_, the computational error was adjusted accordingly (eq 6)­

6 $$\varepsilon_{fit}=\varepsilon_{comp}\cdot m$$

The Kratky plots (*I*_Kratky_) were obtained via the Kratky transformation^102,103^ on the scattering intensity *I* (eq 7)­

7 $$I_{Kratky}=I\cdot q^{2}$$

#### 4.2.5 Chemical Shifts

Backbone amide ^15^N and ^1^H^N^ chemical shifts were predicted using SPARTA+^46^ from individual simulation frames extracted as pdb files by Gromacs trjconv (omitting the first 200 ns).

Mean chemical shifts of single simulations were calculated and further averaged across all trajectories of each MD model before comparison against experimental NMR data.^28^

Errors (ε) were computed by considering the simulation error (ε_sim_), determined as SEM over the 10 trajectories, and the reported prediction error (ε_pred_) of SPARTA+ with 0.49 ppm for ^1^H^N^ and 2.45 ppm for ^15^N^46^

8 $$\varepsilon =\sqrt{\varepsilon_{sim}^{2}+\varepsilon_{pred}^{2}}$$

#### 4.2.6 Spin Relaxation Times

Backbone ^15^N spin relaxation times, T_1_, T_2_, and hetNOEs, were calculated for each residue by Redfield equations^49,50^ as described previously.^13^

Briefly, the spectral density

9 $$J\left(\omega \right)=2\int_{0}^{\infty }C\left(t\right)\cdot \cos\left(\omega \cdot t\right)dt$$

was calculated as the Fourier transformation of the second order rotational correlation function *C*(*t*) of the N–H bond vector

10 $$C\left(t\right)={\left\langle \frac{3}{2}\cos^{2}\left(\theta_{t^{\prime }+t}\right)-\frac{1}{2}\right\rangle }_{t^{\prime }}$$

where θ_*t*′+*t*_ is the angle between the N–H bond vectors at times *t*′ and *t*′ + *t*, and the angular brackets refer to the ensemble average.

The trajectories (omitting the first 200 ns) were processed using the trjconv tool of Gromacs^60^ with the option -pbc nojump. The correlation function *C*(*t*) (eq 10) of each backbone N–H bond was then computed by the Gromacs rotacf tool.^60^ The spectral density *J*(ω) (eq 9) was obtained from *C*(*t*) via regularized inverse Laplace transformation:^13,104^ *N* = 1000 exponential functions were fitted according to

11 $$C\left(t\right)=\sum_{i=1}^{N}\alpha_{i}\cdot e^{-t/\tau_{i}}$$

where rotational time scales τ_*i*_ are logarithmically spaced in the range from 10^–1^ to 10^5^ ps and the related weight coefficients are represented by α_*i*_.

The averages of T_1_, T_2_, and hetNOEs across all trajectories of an individual MD model were compared to data from NMR experiments at field strengths of 600 and 850 MHz. Errors were determined as SEM over the 10 trajectories.

#### 4.2.7 Backbone Orientational Correlation of Cα Vectors

Analysis of backbone orientational correlations was done according to Virtanen et al.:^51^ For residues *i* and *j*, we calculated the average dot product $\left\langle \vec{v_{i}}\cdot \vec{v_{j}}\right\rangle$ , where $\vec{v_{i}}$ is the unit vector from Cα_i_ to Cα_*i*+1_, with the average taken over the trajectory. A positive or negative result indicates an orientational correlation. Random vector orientations lead to an average value of zero—as do vectors that are consistently orthogonal to each other since the dot product is zero in that case.

#### 4.2.8 Conditions in Experiments and Simulations

COR15A is a strong polyampholyte due to its high fraction of charged residues, very low net charge per residue, and nearly perfect charge mixing^25^ (Figure S22). This low charge segregation minimizes long-range electrostatic interactions.^105^ We therefore assume that minor ionic strength differences between simulations and experiments (Table 2) should not substantially impact the results.

**Table 2:** Conditions in Experiments and Simulations^a^

system	buffer	pH	temperature	protein net charge
SAXS	10 mM Na_2_HPO_4_/NaH_2_PO_4_	7.4	room temperature	–6
NMR	18 mM Na_2_HPO_4_/NaH_2_PO_4_	7.0	298.16 K	–6
CD	10 mM Na_2_HPO_4_/NaH_2_PO_4_	7.4	296.16 K	–6
simulation	water	7.0	300 K	–6

a All MD systems include six sodium ions to ensure net charge neutrality and constant protonation states mimicking those at pH 7.0.

The protein net charge for the experimental systems (Table 2) was determined using the H++ online server^106^ (version 4.0), which calculates residue-specific intrinsic p*K* values from computed titration curves by considering the specified pH and salinity.

### 4.3 Protein Expression and Purification

^15^N labeled COR15A WT and G68A were prepared from pProEX HTb plasmids as previously described,^28^ facilitating purification via a HIS_6_-tag, which was cleaved by a TEV protease subsequently. Briefly, plasmids were expressed in *Escherichia coli* BL21­(DE3) cells in M9 medium supplemented with ^15^N ammonium chloride (Sigma-Aldrich, Taufenkirchen, Germany) at 37 °C until an OD_600_ of 0.6. Recombinant gene expression was induced with 1 mM isopropyl-β-d-1-thiogalactopyranoside (IPTG), and cells were grown overnight. After cell harvest by centrifugation, resuspension in 20 mM Na_2_HPO_4_/NaH_2_PO_4_ pH 7.0, 500 mM NaCl, and lysis by sonication, recombinant ^15^N-COR15A WT and ^15^N-G68A were purified from the supernatant by affinity chromatography using a 5 mL HisTrap column (Cytiva, Marlborough, MA, US) under gradient elution with 20 mM Na_2_HPO_4_/NaH_2_PO_4_ pH 7.0, 500 mM NaCl, 250 mM imidazole. The HIS_6_-tag was cleaved by a custom-made TEV protease, and the cleaved HIS_6_-tag and the similarly HIS_6_-tagged TEV protease were separated from untagged ^15^N-COR15A WT and ^15^N-G68A by a second affinity chromatography. Proteins were dialyzed against ddH_2_O and lyophilized.

### 4.4 NMR Experiments

Samples for NMR measurements were prepared as follows: ^15^N-COR15A WT aliquot of 1.9 mg and ^15^N-G68A aliquot of 0.75 mg were dissolved in 600 μL of 20 mM Na_2_HPO_4_/NaH_2_PO_4_ pH 7.0 with 10% D_2_O. For both protein variants, two independent samples were prepared. While ^15^N-COR15A WT remained stable for several months, ^15^N-G68A began to aggregate after approximately 75 h. As a result, this sample could not be used for measurements at different magnetic fields.

All NMR spectra were recorded at 298 K. The two ^15^N-COR15 WT samples and ^15^N-G68A sample 1 were measured using a Bruker Avance III 600 MHz spectrometer (^1^H frequency: 597.427 MHz) equipped with a 5 mm double-resonance (X, ^1^H) broadband probehead (SmartProbe) with *Z*-axis gradients. ^15^N-COR15 WT sample 2 and ^15^N-G68A sample 2 were measured on a Bruker Avance IIIHD 850 MHz spectrometer equipped with a cryogenically cooled probe at the Institute of Biotechnology, University of Helsinki.

Peaks obtained from HSQC-based experiments were assigned based on previous data.^28^

In the 600 MHz spectrometer, spin relaxation times were acquired with a HSQC-based pulse sequence^50,107,108^ using parameters listed in Table 3. Delay times were set to 20, 50, 100, 200, 300, 500, 700, 900, 1500, and 1900 ms for T_1_ and 34, 51, 68, 85, 119, 153, 187, 220, and 254 ms for T_2_. A recycling delay of 3.5 s between scans was used for both T_1_ and T_2_, and 5 s for heteronuclear NOE. HetNOE was measured with 16 scans.

**Table 3:** NMR Measurements

sample	magnetic field (MHz)	experiment	F3 points	F2 points	F1 points	number of scans
WT 1	600	T_1_	1450	223	10	8
WT 1	600	T_2_	1450	300	9	8
WT 1	600	hetNOE		1450	600	16
WT 2	600	T_1_	1450	300	10	8
WT 2	600	T_2_	1450	300	9	8
WT 2	600	hetNOE		1450	600	16
G68A 1	600	T_1_	1450	250	10	8
G68A 1	600	T_2_	1450	300	9	8
G68A 1	600	hetNOE		1450	431	16
WT 1	850	T_1_	2048	250	10	8
WT 1	850	T_2_	2048	250	9	8
WT 1	850	hetNOE		2048	512	32
G68A 1	850	T_1_	2048	250	10	8
G68A 1	850	T_2_	2048	250	9	8
G68A 1	850	hetNOE		2048	512	32

In the 850 MHz spectrometer, temperature-compensated versions of T_1_ and T_2_ HSQC-based pulse sequences were used,^108^ with parameters listed in Table 3. The delay times were the same as those used at 600 MHz. Also, details for hetNOE experiments are given in Table 3.

The T_1_ and T_2_ relaxation data were processed using TopSpin software (version 3.6, Bruker), and the decay of peak heights with increasing delay times was analyzed by using a Python script with monoexponential decay fitting. Experimental errors were estimated from the fitting error, except for the WT measurements at 600 MHz, where errors were determined as the SEM from two independent measurements. For the analysis of hetNOE values, peak heights were determined by TopSpin software from spectra with and without NOE. To determine the errors for hetNOEs, the signal-to-noise ratio was first determined from a region without any peaks, and the limiting extremes of the noise values were then added to or subtracted from the peaks to estimate the maximal noise effect on the ratio between the peaks in the two spectra.

## Supplementary Material



## Notes

Additional information on generation of initial conformational ensembles; further detailed simulation results and analyses; further experimental details of COR15A WT and G68A; and further details on the dependence of relaxation parameters on the time scale of molecular motion (PDF)
