# Supplementary material for: Molecular Dynamics of the Intrinsically Disordered Protein COR15A—A Force Field Validation on Structure and Dynamics
Source: J Chem Theory Comput. 2025 Sep 4;21(18):9147–63. doi: 10.1021/acs.jctc.5c00854 (PMC12461933; doi:10.1021/acs.jctc.5c00854)
Supplement: Supplementary file 1 [file ct5c00854_si_001.pdf]

Supplementary: Molecular dynamics of the intrinsically disordered protein COR15A – A force field validation on structure and dynamics

Tobias Rindfleisch,[†,‡,¶,§] Ricky Nencini,[‖,⊥] O. H. Samuli Ollila,[#,‖] Dirk Walther,[§] Markus S. Miettinen,[*,‡,†,@] and Anja Thalhammer[*,¶]

†*Computational Biology Unit, Department of Informatics, University of Bergen, Bergen, Norway*
‡*Department of Chemistry, University of Bergen, Bergen, Norway*
¶*Physical Biochemistry, University of Potsdam, Potsdam, Germany*
§*Max-Planck Institute of Molecular Plant Physiology, Potsdam, Germany*
‖*Institute of Biotechnology, University of Helsinki, Helsinki, Finland*
⊥*Division of Pharmaceutical Biosciences, Faculty of Pharmacy, University of Helsinki, Helsinki, Finland*
#*VTT Technical Research Centre of Finland, 02044 Espoo, Finland*
@*Department of Theory and Bio-Systems, Max Planck Institute of Colloids and Interfaces, Potsdam, Germany*

E-mail: markus.miettinen@iki.fi; anja.thalhammer@uni-potsdam.de

Data availability

The MD trajectory files of all simulations performed in this study and the corresponding scripts for system set-up, MD production runs and analysis of results are available under:

`https://doi.org/10.17617/3.4I538Z`

S1 Supplementary Information

Generation of initial conformational ensembles

We modeled the initial conformations for the COR15A WT and G68A ensembles using a *de novo* approach by performing ten 150 ns MD simulations with independent initial velocities for each COR15A variant in aqueous systems. The trajectories of both proteins reached characteristic conformational sampling after about 80 ns, as monitored by the averaged root mean square deviation (RMSD), while smaller fluctuations are in agreement with the presumed disordered nature of COR15A (Figure S25). The amplitudes and shapes of RMSD curves are similar for both COR15A variants, implying that the single substitution in the G68A mutant does not affect the kinetics of reaching a structural equilibrium and the overall protein fluctuations.

Because of the flexible behavior of IDPs, the heterogeneity and independence of the conformations representing the initial structural ensemble is of special importance. To ensure heterogeneity and to avoid the oversampling of extreme states, we plotted the distributions of the radius of gyration (R_G) per individual trajectory, sampled from the equilibrium state, for COR15A WT and G68A in Figure S26. For both protein variants, structures with R_G between about 12 and 35 Å were modeled and no major differences were observed between the variants. Our ensembles thus consisted of heterogeneous sets of conformations, spanning trajectories with diverse structures with a large extension, (Figure S26A), indicating disordered and flexible conformations (e.g. WT-4, WT-8, G68A-6 and G68A-10) (Figures S23D,H, S24F,J), to ones with narrow R_G distributions indicating the presence of compact structures with lower flexibility (WT-3, WT-6, G68A-3) (Figures S23C, S24F,J). The results ensure that a large heterogeneity of conformations in agreement with the properties of IDPs was sampled.

Subsequently, we evenly extracted 25 conformations from each initial trajectory in equilibrium, resulting in 250 structures per COR15A variant. These models were clustered with the aim to assign the centromere structures of the identified group as members of the initial conformational ensemble for further analysis.

The difference and thus the distance between a pair of structures is illustrated in Figure S27 as heat maps based on the TM score. The score of structures corresponding to different trajectories is rather low and in most cases smaller than 0.17, which is typical for randomly chosen protein pairs.[1] Scores >0.5 are characteristic for structure pairs with similar folds[1] and are only observable within the single clusters. The only two clusters with lower scores (WT-8 and G68A-10) (Figure S27) contain extended IDP conformations (Figure S26) which are more affected by the fluctuation of flexible substructure elements than more compact polypeptides. Thus, the higher protein mobility is likely associated with a lower similarity of the inherent cluster structures. On the other hand, trajectories with more compact conformations, as e.g. trajectory WT-2 and G68A-3 (Figure S26) show a significantly higher inherent cluster similarity (Figure S27). Together, these results indicate a correlation between the degree of extension for inner-cluster conformations and the corresponding inherent cluster similarity, likely because in proteins with a higher compactness, the intramolecular and mostly distance-dependent interactions like H-bonds and electrostatic interactions are much more pronounced. In both distance matrices, we identified ten independent clusters, each consisting of the models extracted from the respective trajectory (Figure S27), demonstrating the independence of the initial MD simulations, which all started from the fully extended chains of COR15A WT and COR15A G68A, respectively. The centromeres of these clusters were selected for the initial ensembles used in all subsequent analyzes. No statistical weighing of the conformations from different clusters included in the conformational ensemble was performed as this would require to determine the accessible phase space and each simulation's sampled region of this phase space. Therefore, unweighted or random sampling of initial conformation is common in the literature.[2,3]

S2 Supplementary Figures

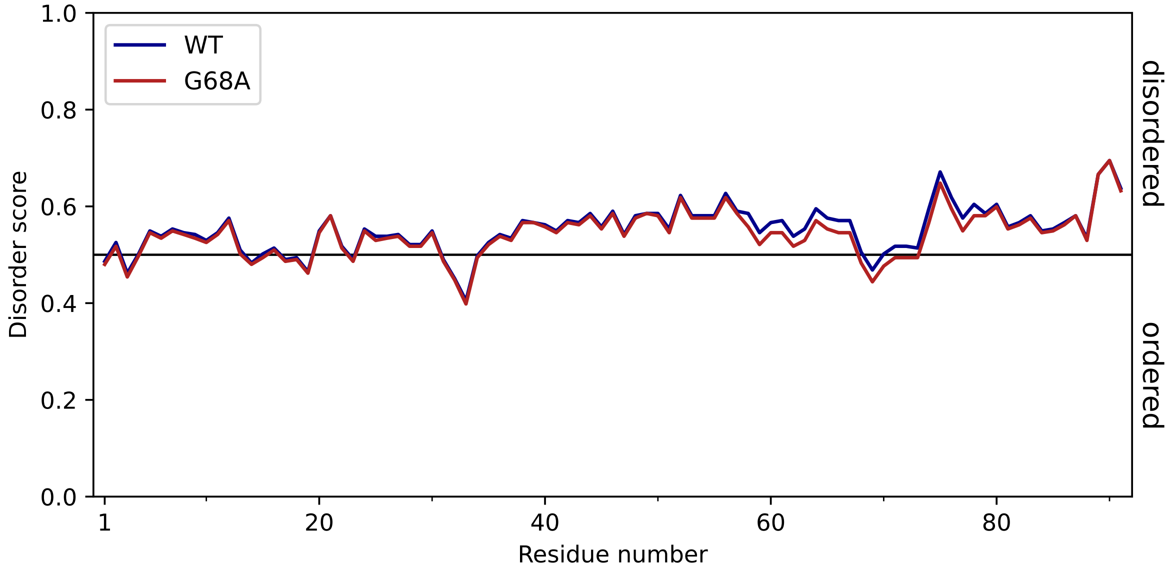

Figure S1: Per-residue disorder score of COR15A WT (blue) and COR15A G68A (red) predicted by the online tool IUPred2A.[4] Scores > 0.5 imply a disordered character of the corresponding protein sequence, scores < 0.5 indicate regions in ordered states.

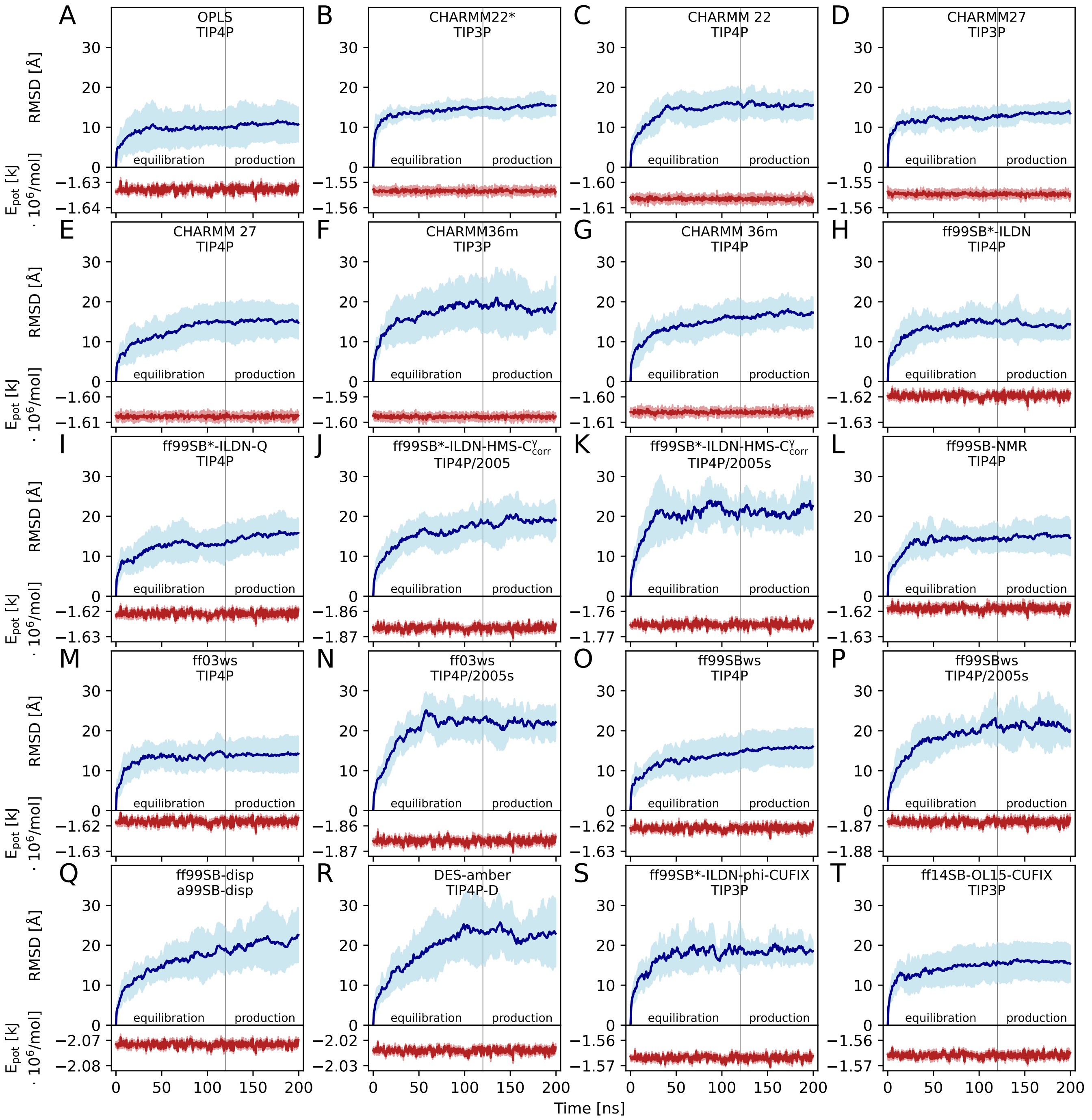

Figure S2: Root mean square deviation (RMSD) and potential energy (E_{pot}) during simulations for force field validation. The upper figure in each subplot demonstrates the development of the RMSD averaged over all members of the ensemble based on the backbone atoms of the protein for the simulation systems listed in Table 1. The lower figure in each subplot represents the averaged potential energy for the corresponding simulation system in the upper subplot. Standard deviations are marked in lighter colours.

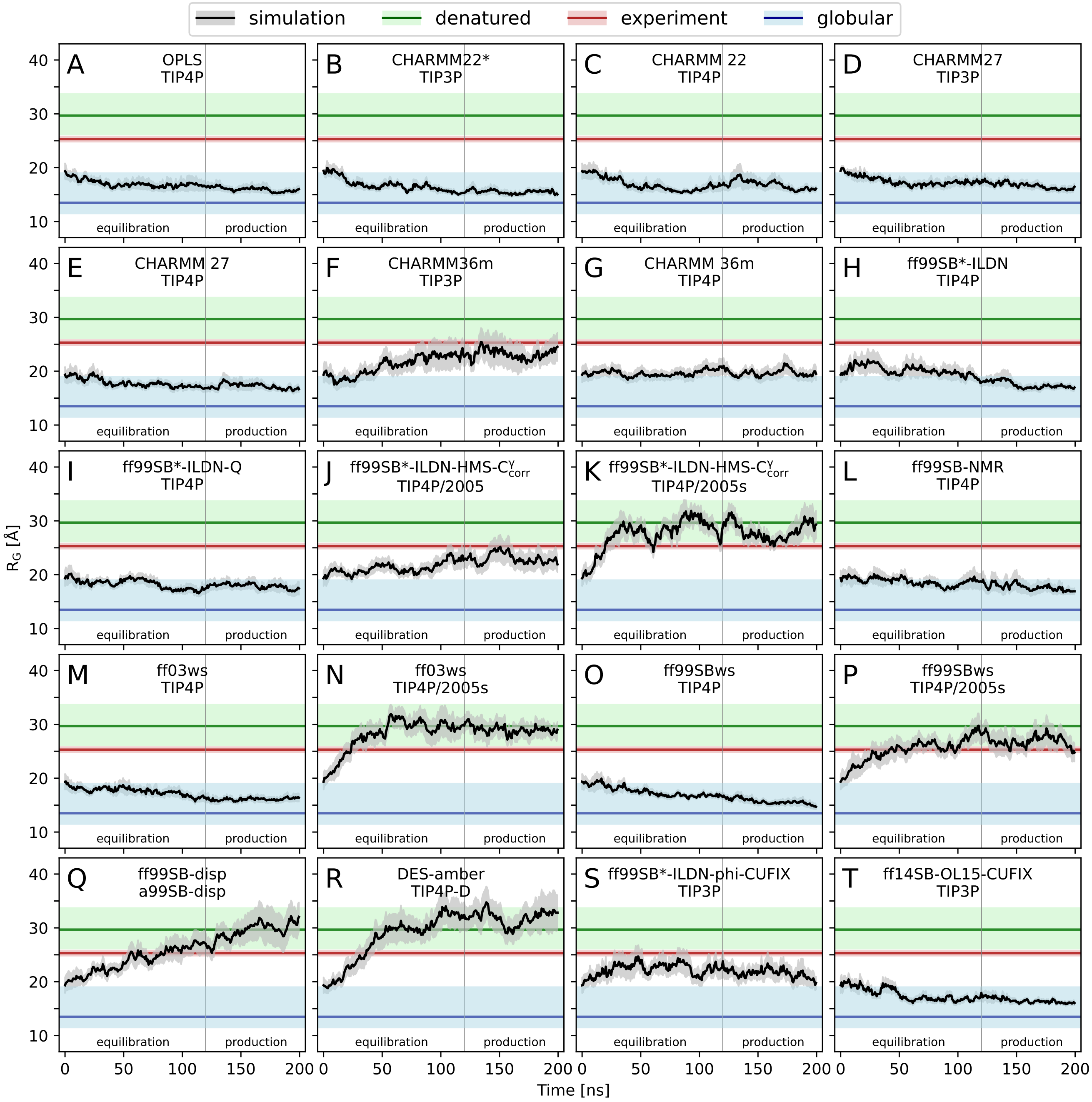

Figure S3: Evaluation of R_G over time for all 20 MD models listed in Table 1 derived from ten replicate simulations. The red line shows the experimental reference value determined by SAXS;[5] R_G values approximated from scaling laws for globular[6] (blue line) and denatured[7] (green line) proteins with molecular weights identical to COR15A WT. Estimated error is visualized in lighter color.

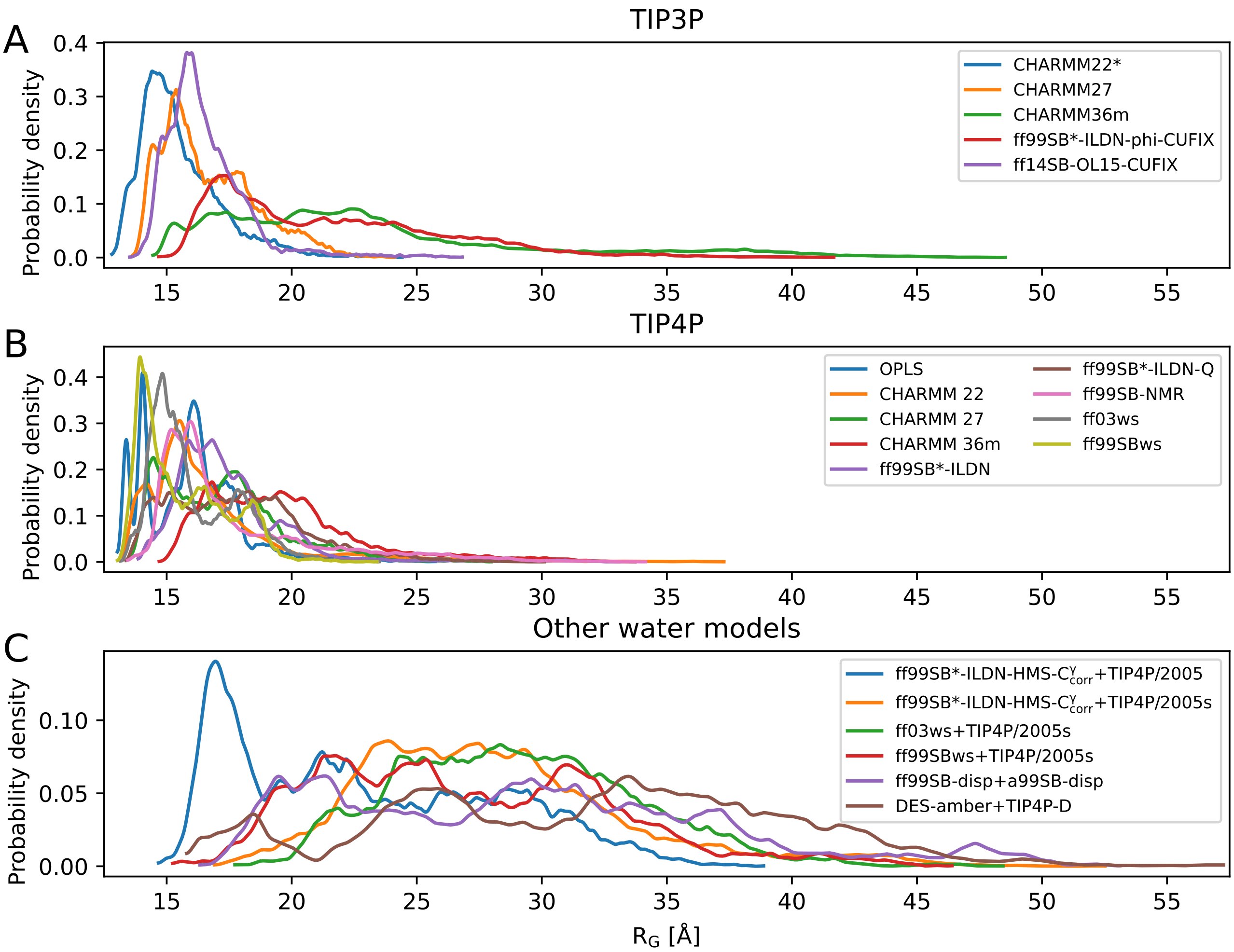

Figure S4: Global distribution of R_G of all individual structures constituting the conformational ensemble for MD systems tested during the force field validation. Systems including the TIP3P or TIP4P water model are shown in **A** and **B**, respectively. Systems using other water models are illustrated in **C**. All probability density distributions are represented by 1000 bins and the resulting curves were slightly smoothed applying a gaussian filter (python package `scipy`[8]) for clearer representation.

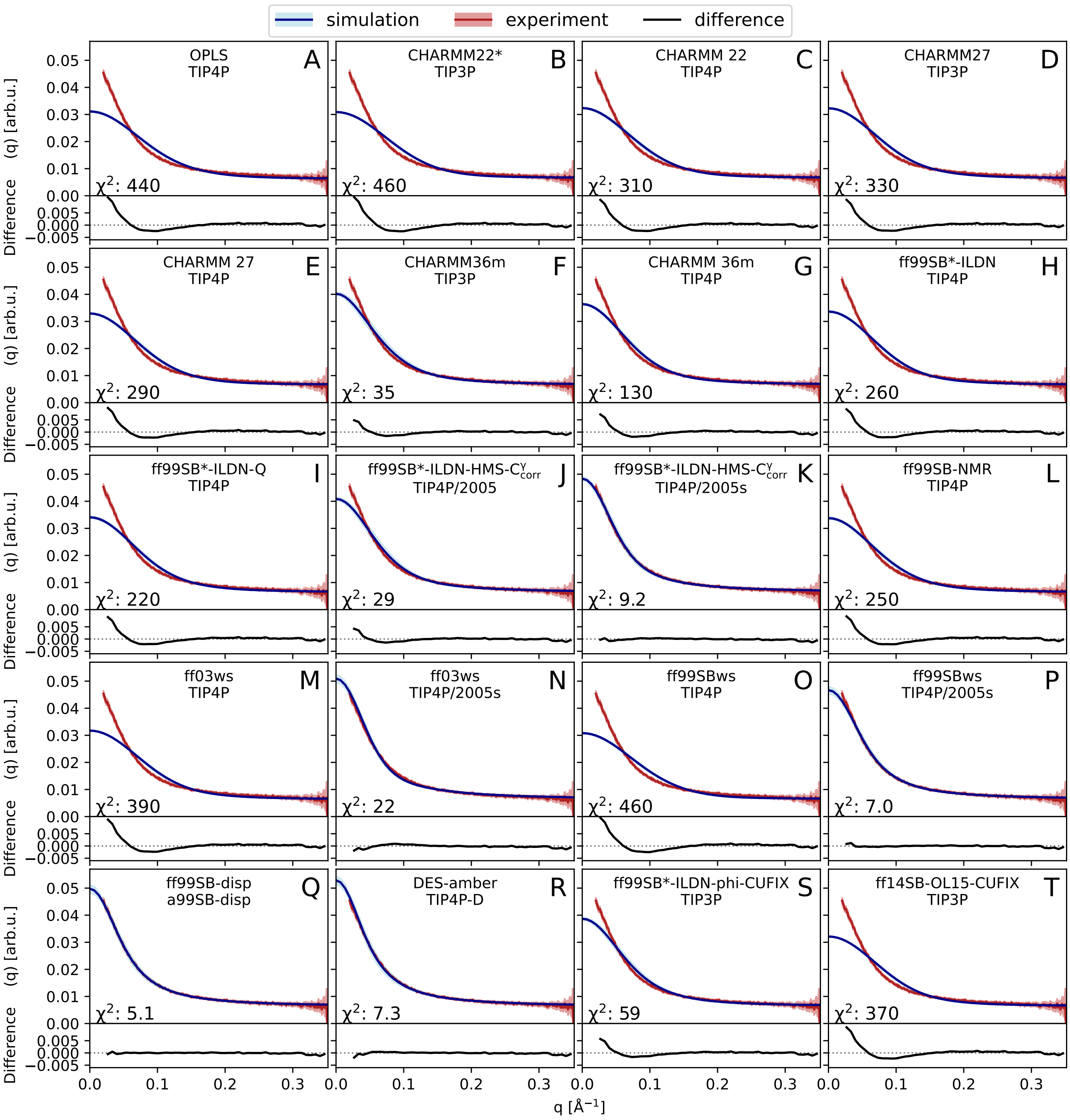

Figure S5: COR15A WT SAXS profiles, each computed from n=10 independent simulations (blue) compared against experiment[5] (red) in lin-lin representation in the upper panel of each subplot for all 20 MD models listed in Table 1 . Experimental errors and the standard errors of the mean (SEM) for the computed profiles are indicated in lighter shades. The χ^2 is between experiment and simulation. The lower panel in each subplot represents the difference (experiment-simulation).

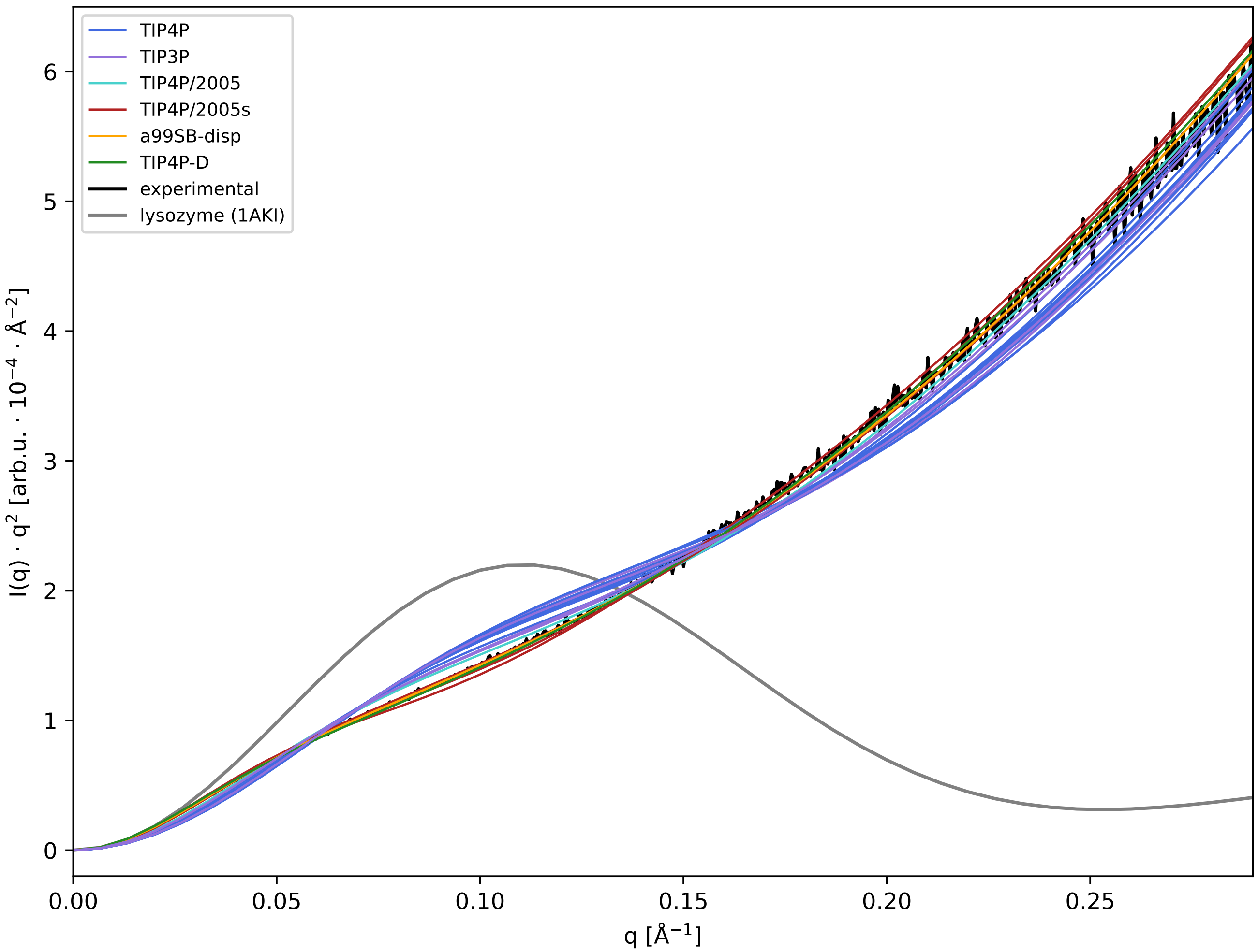

Figure S6: Kratky transformations of the computationally determined SAXS scattering profiles for all MD systems listed in Table 1 in comparison to experimental data (black) and the Kratky plot of the folded protein hen egg-white lysozyme (PDB: 1AKI) (grey) for visualizing the difference between ordered and disordered proteins. The computed graphs are colour-separated by the water models.

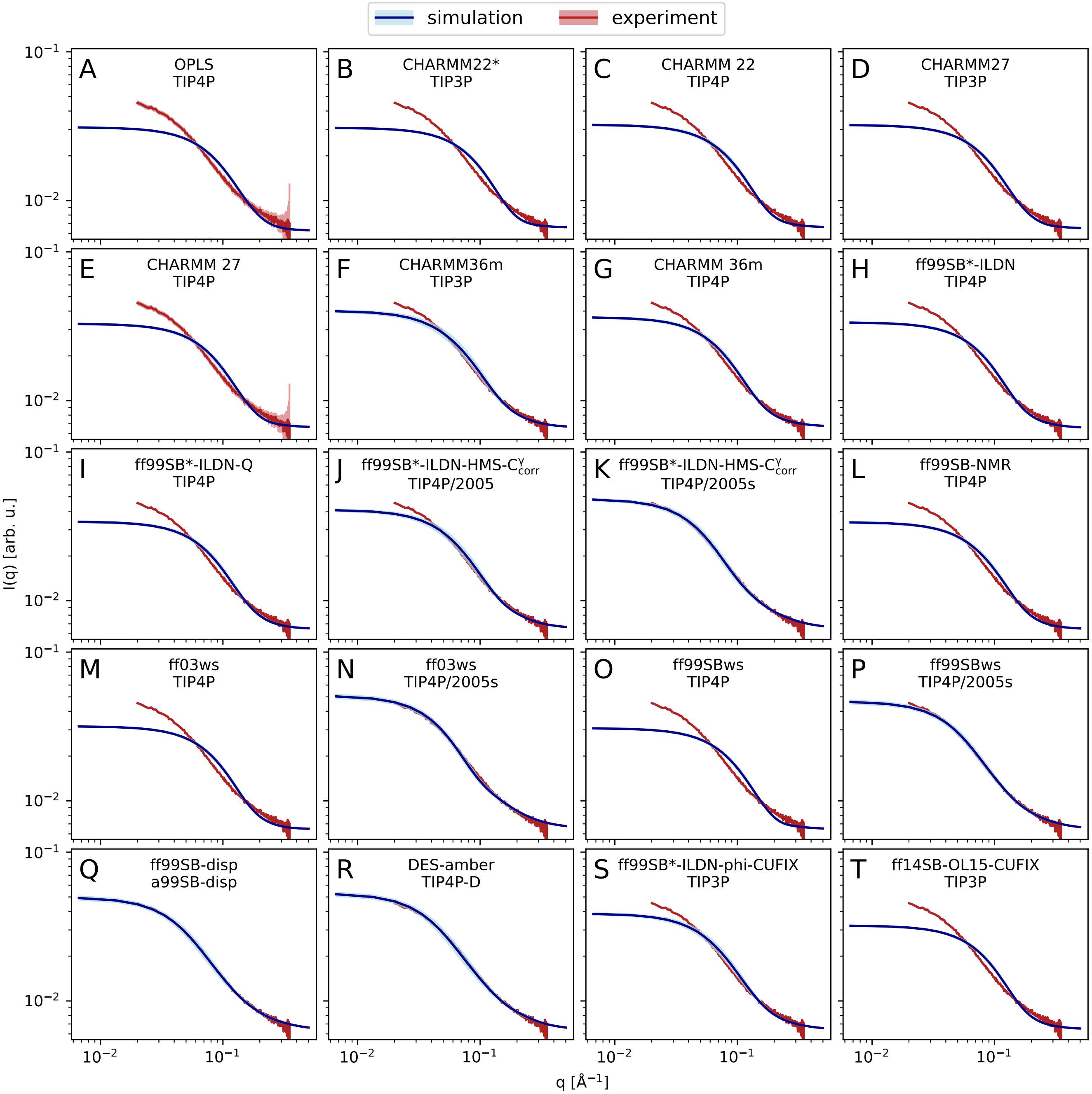

Figure S7: COR15A WT SAXS profiles, each computed from n=10 independent simulations (blue) compared against experiment[5] (red) in log-log representation in the upper panel of each subplot for all 20 MD models listed in Table 1 . Experimental errors and the standard errors of the mean (SEM) for the computed profiles are indicated in lighter shades.

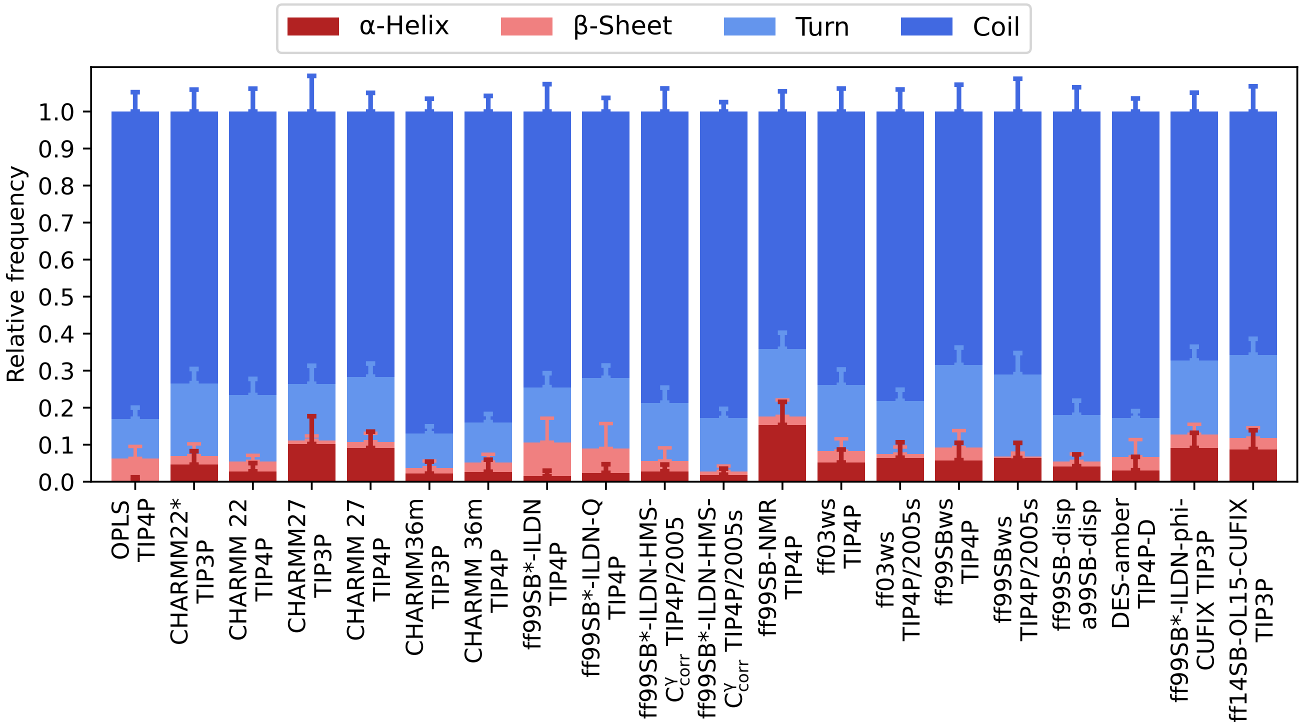

Figure S8: Mean secondary structure composition of COR15A WT for all 20 MD models listed in Table 1. Global secondary structure of all individual structures constituting the conformational ensemble was calculated using the DSSP algorithm[9,10] and averaged over all single MD simulation frames. The averaged proportions of the secondary structure elements α-Helix (red), β-Sheet (light red), Turn (light blue) and Coil (blue) are illustrated as stacked bar plots with error bars representing the standard deviation.

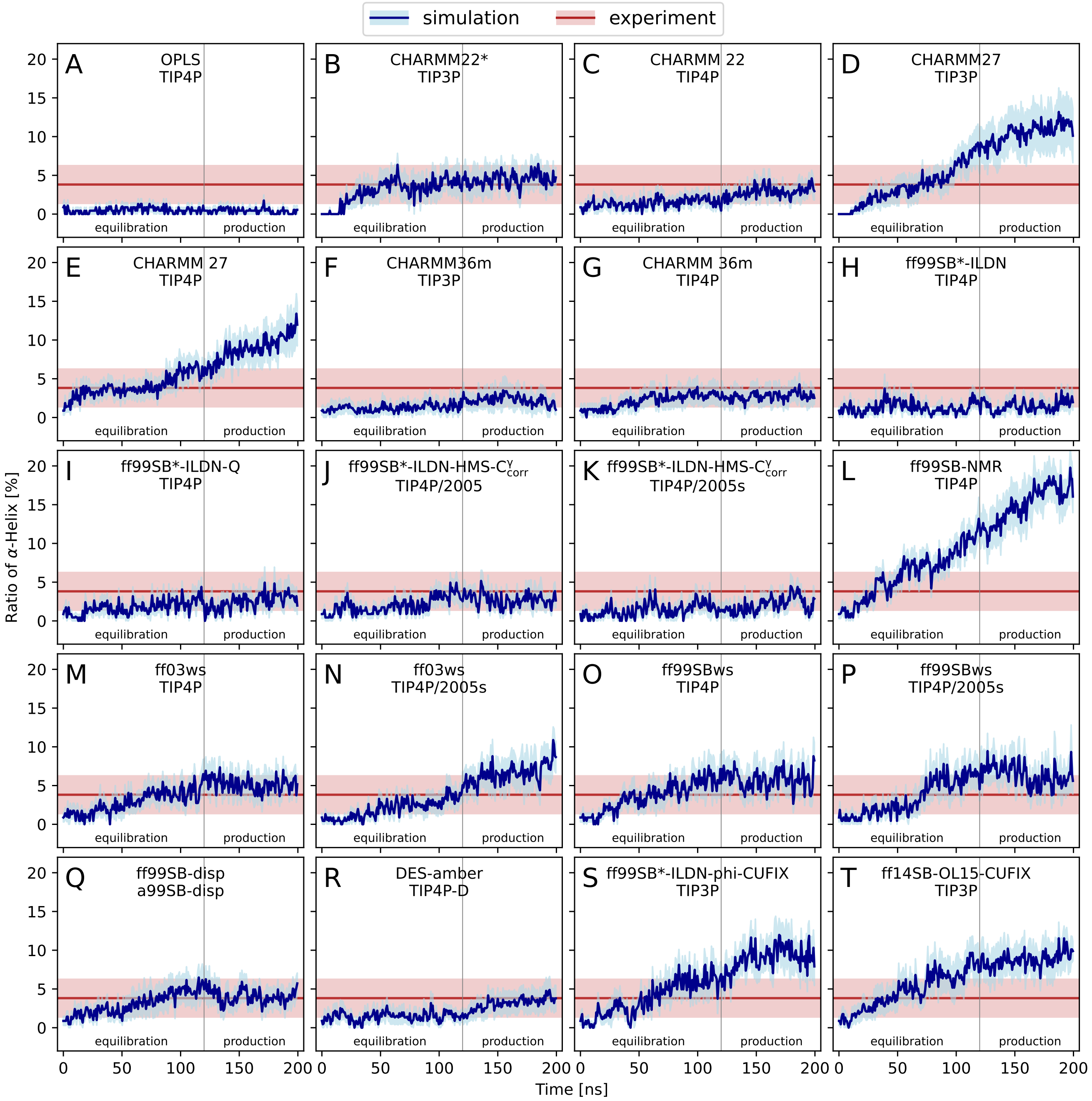

Figure S9: Evaluation of α-helicity over time for all 20 MD models listed in Table 1 derived from ten replicate simulations with shaded areas representing the standard error of the mean (SEM). The red line shows the per residue averaged experimental reference determined from NMR chemical shift analysis[11] with corresponding standard deviation as estimated error visualized in lighter color.

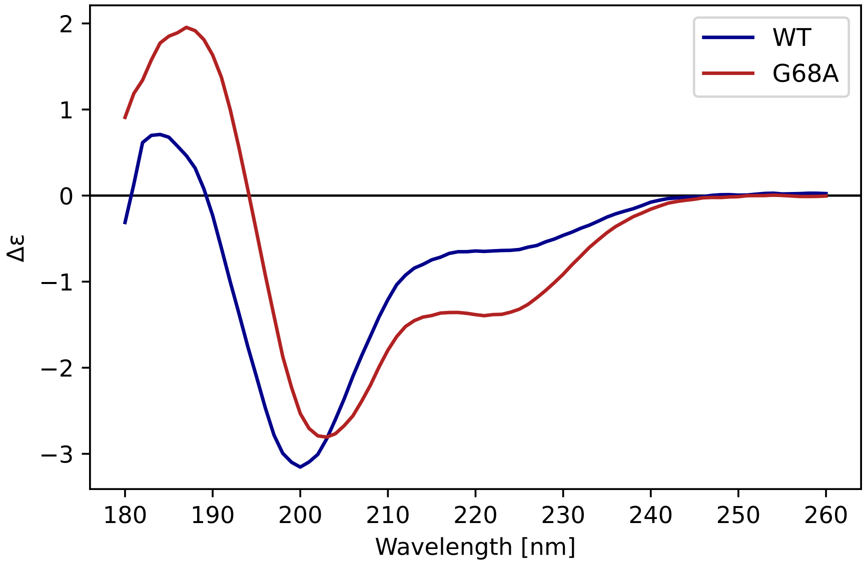

Figure S10: CD spectra of COR15A WT and its mutant G68A in 20 mM sodium phosphate buffer scaled from.[11]

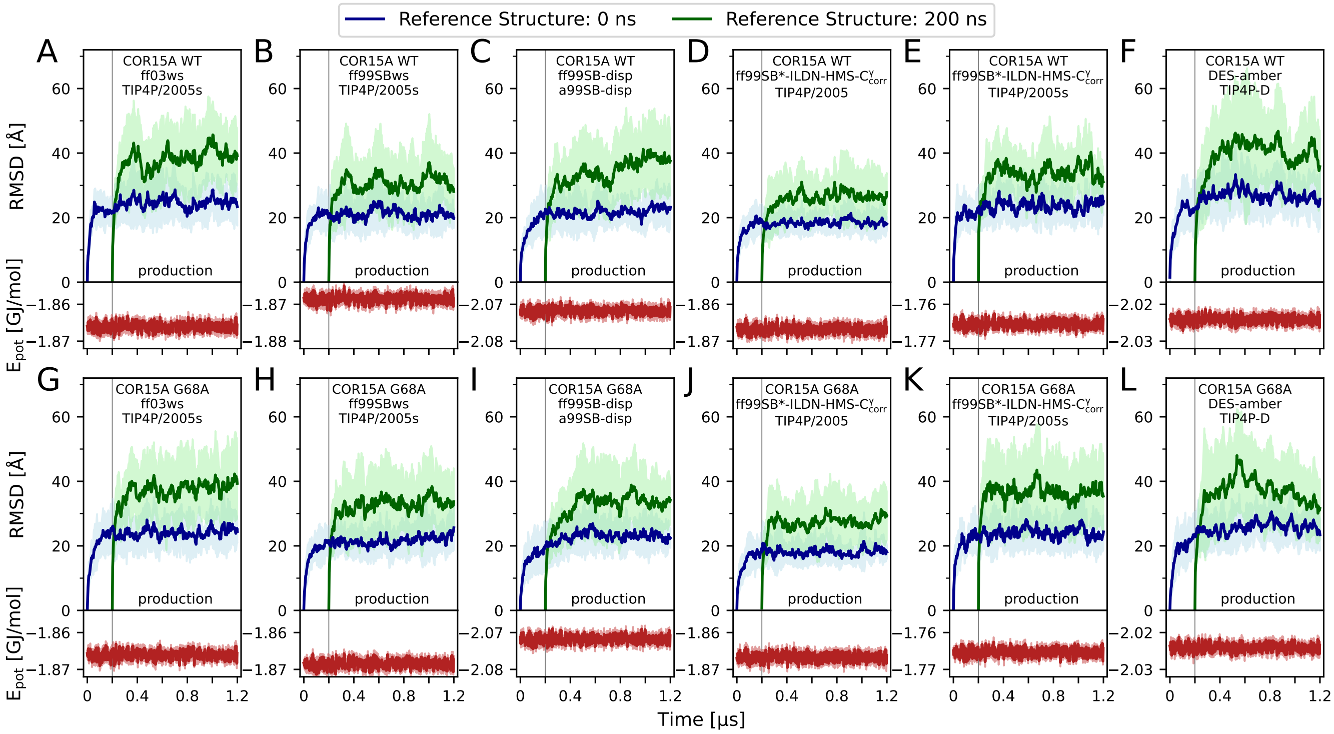

Figure S11: The upper figure in each subplot shows the development of the RMSD as function of time (t), averaged over all 1.2 µs simulations, using the conformations at 0 ns (blue) and 200 ns (green) as references. RMSD relative to t = 0 ns reflects differences in conformational space sampling between OPLS-AA, used during initial ensemble generation, and the referenced MD systems. The RMSD relative to t = 200 ns captures conformational variation within the sampling of each of the six selected MD systems. The latter RMSD values are consistently larger than those referenced to t = 0 ns because the related initial OPLS-AA ensemble consists of compact structures, while the equilibrated MD systems at t = 200 ns adopt more extended conformations; the average RMSD between a compact and extended conformation is less than between two distinct extended conformations. RMSD was calculated based on the backbone atoms of COR15A WT (**A**-**D**) and G68A (**E**-**H**). The standard deviation is marked in lighter colours. The lower figure in each subplot represents the averaged potential energy for the corresponding simulation system. The first 200 ns of the simulation were considered as extended equilibration; the remaining time was used for production analysis.

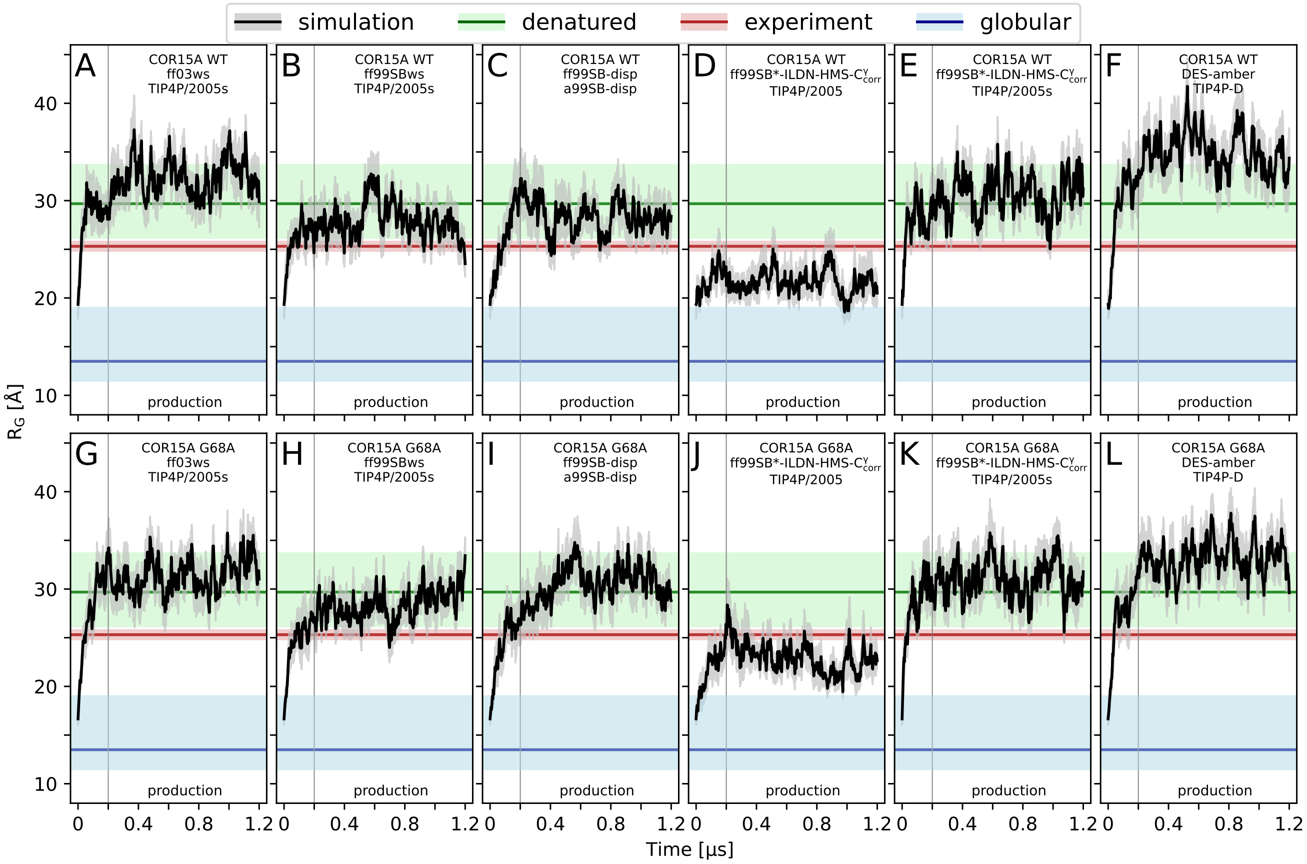

Figure S12: Evaluation of R_G over time for six MD models derived from ten replicate simulations of COR15A WT and G68A, respectively. The red line shows the experimental references determined by SAXS;[5] R_G values approximated from scaling laws for globular[6] (blue line) and denatured[7] (green line) proteins with molecular weights identical to COR15A WT. Estimated error is visualized in lighter color. The first 200 ns of the simulation were considered as extended equilibration; the remaining time was used for production analysis.

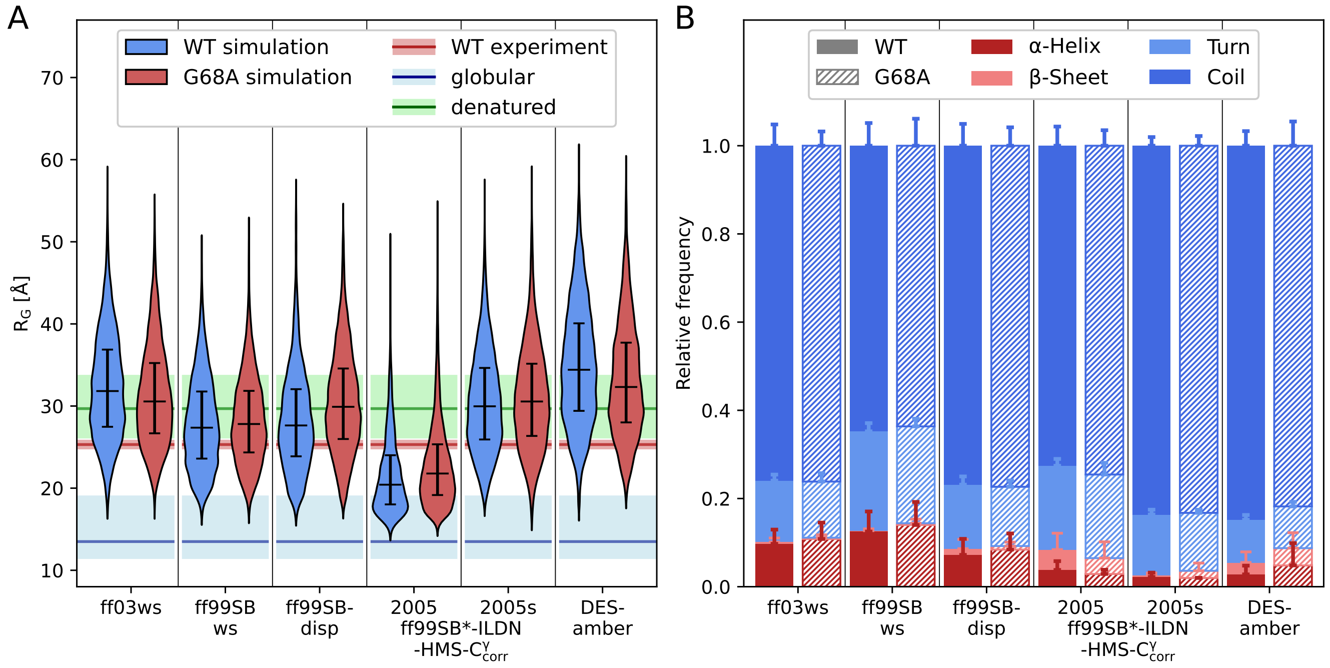

Figure S13: The violins in **A** show the R_G distribution across all conformations sampled in the ten equilibrated 1-μs replicate simulations for each MD system in context to the experimental data (red line) and to R_G of globular[6] (blue line) and denatured[7] (green line) proteins with molecular weights identical to the one of COR15A WT derived from scaling laws. The estimated error of each reference is marked as area in lighter color. In the violins, long dashes represent the median, error bars the 25% and 75% quartiles . **B** represents the averaged proportions of the secondary structure elements α-Helix (red), β-Sheet (light red), Turn (light blue) and Coil (blue) for the systems above as stacked bars. Error bars represent the standard deviation.

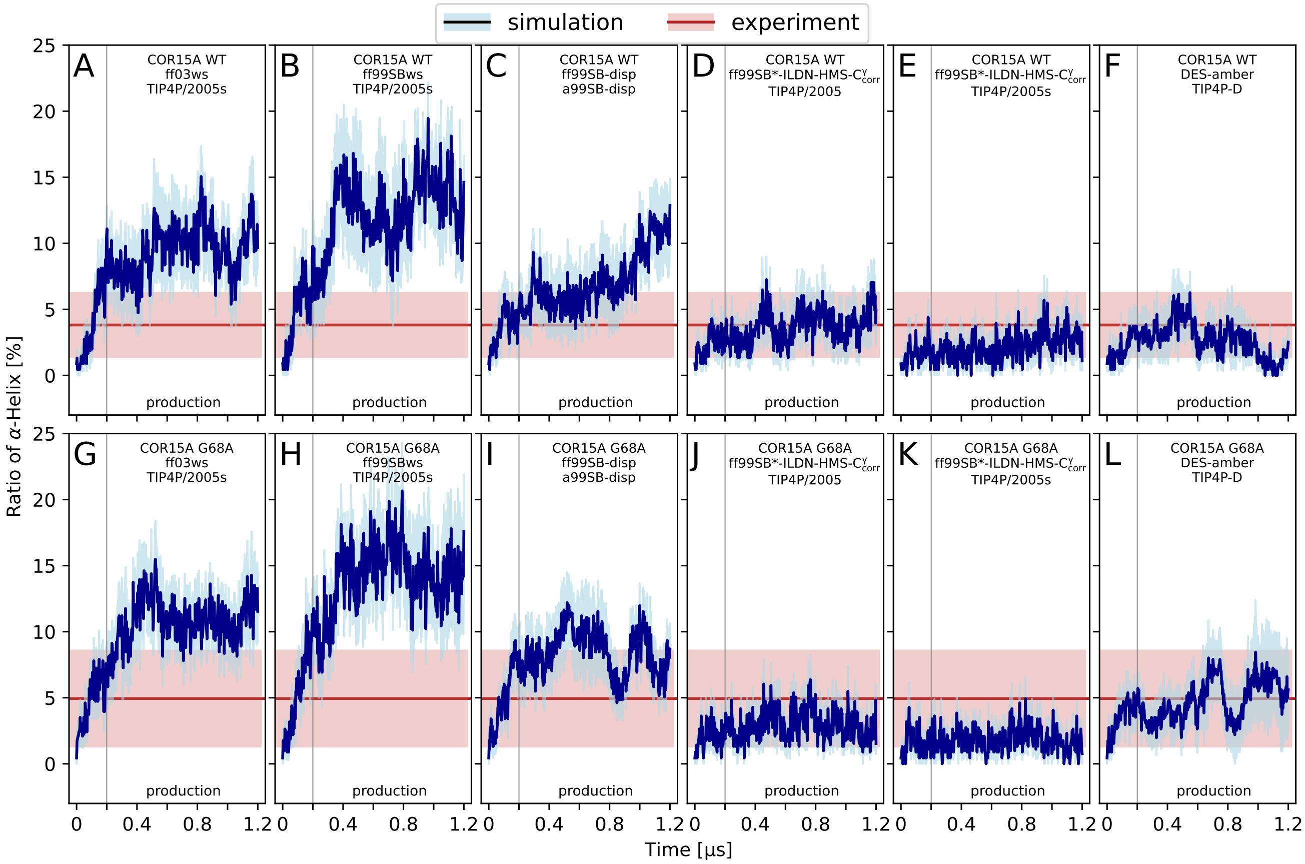

Figure S14: α-helicity over time was averaged over all trajectories of a MD system and is shown for COR15A WT (**A**-**F**) and G68A (**G**-**L**) for the MD systems ff03ws, ff99SBws, ff99SB-disp, ff99SB*-ILDN-HMS-C^{γ}_{corr} in combination with the water models TIP4P/2005 and TIP4P/2005s and DES-amber as function of time. The red line shows the experimental references determined from NMR chemical shift analysis[11] with corresponding error visualized in lighter color. The first 200 ns of the simulation were considered as extended equilibration; the remaining time was used for production analysis.

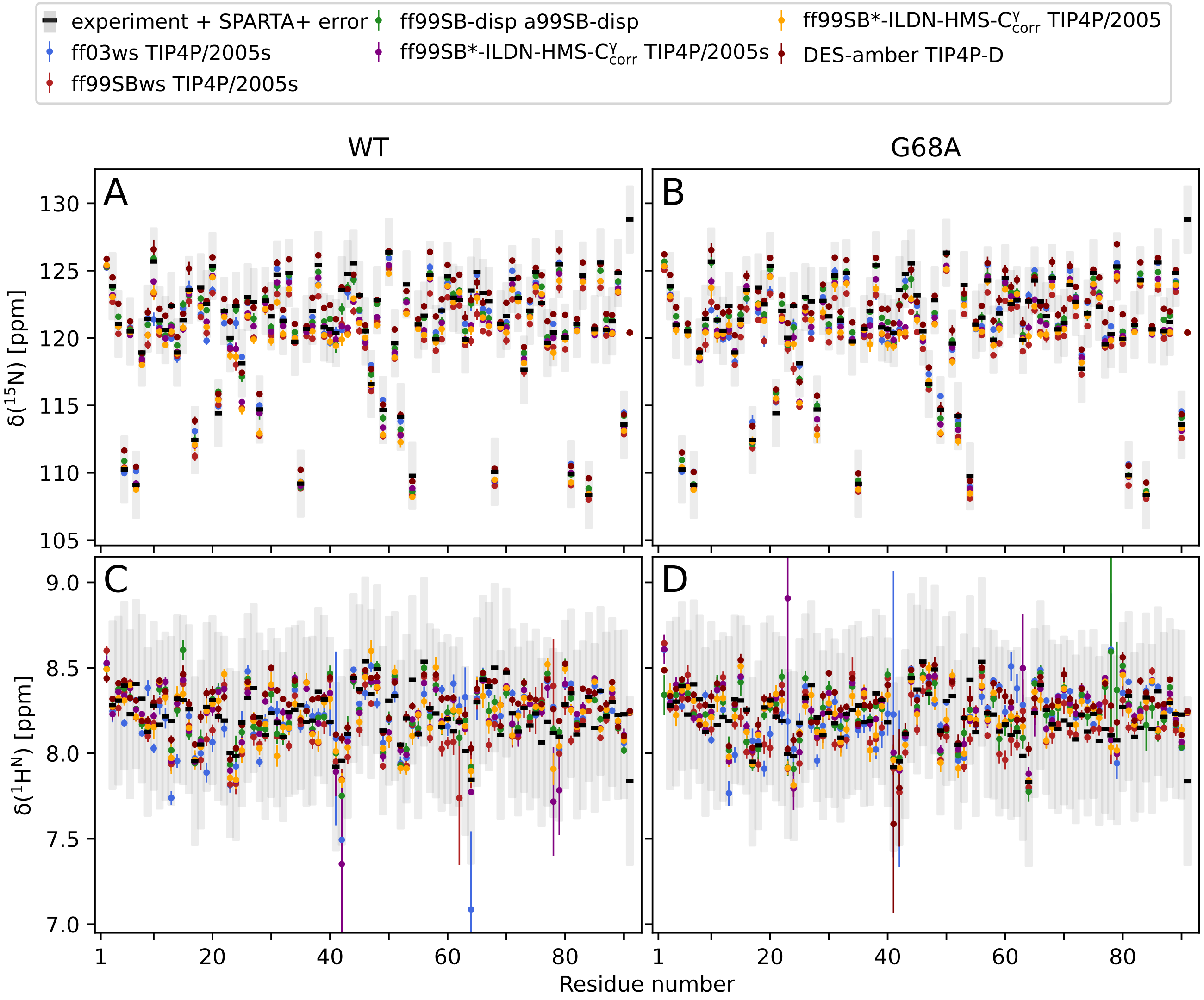

Figure S15: Plots of backbone amide ^{15}N (**A**,**B**) and ^{1}H^{N} (**C**,**D**) chemical shifts (δ) derived from experiments and predicted from simulation frames via SPARTA+[12] are shown for COR15A WT and G68A for the MD systems ff03ws, ff99SBws, ff99SB-disp, ff99SB*-ILDN-HMS-C^{γ}_{corr} in combination with the water models TIP4P/2005 and TIP4P/2005s and DES-amber. Gray bars represent the prediction error of SPARTA+—0.49 ppm for ^{1}H^{N} and 2.45 ppm for ^{15}N[12]—which was added to the experimental values. The simulation error was calculated as the standard error of the mean (SEM) from n=10 independent simulations.

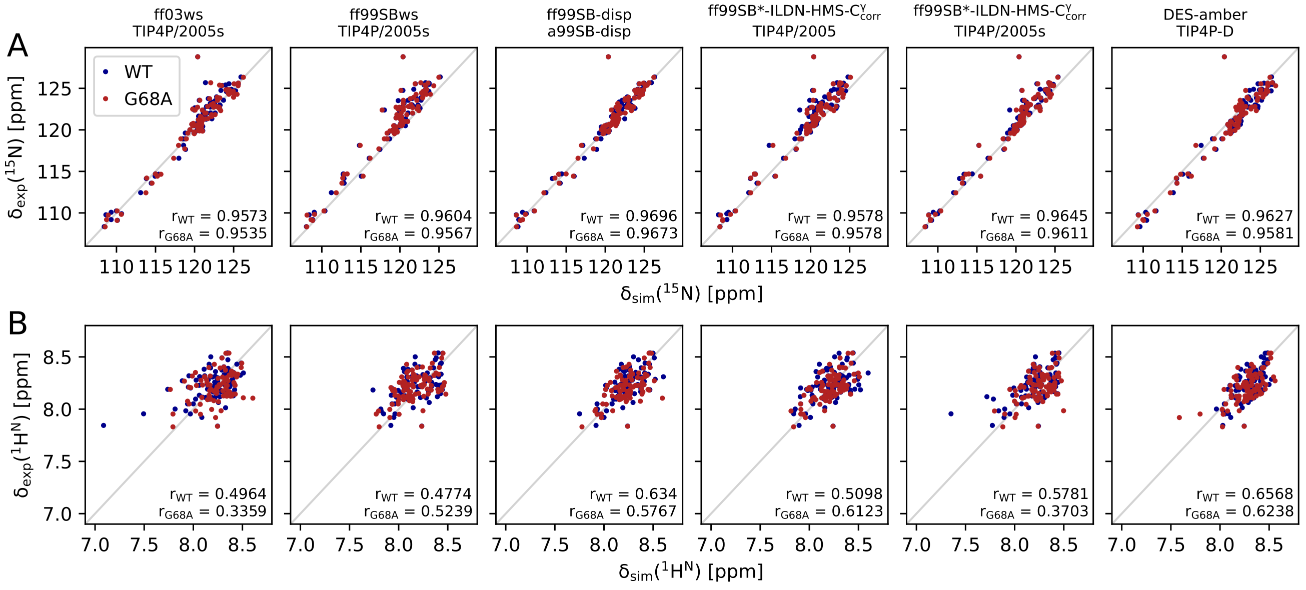

Figure S16: Scatter plots of backbone amide ^{15}N (**A**) and ^{1}H^{N} (**B**) chemical shifts derived from experiments[11] (δ_{exp}) and predicted (δ_{pred}) from simulation frames via SPARTA+[12] are shown for COR15A WT (blue) and G68A (red) for the MD systems ff03ws, ff99SBws, ff99SB-disp, ff99SB*-ILDN-HMS-C^{γ}_{corr} in combination with the water models TIP4P/2005 and TIP4P/2005s and DES-amber. In the individual scatter subplots, the correlation between δ_{exp} and δ_{pred} is reported as r_{WT} and r_{G68A} for COR15A WT and G68A, respectively.

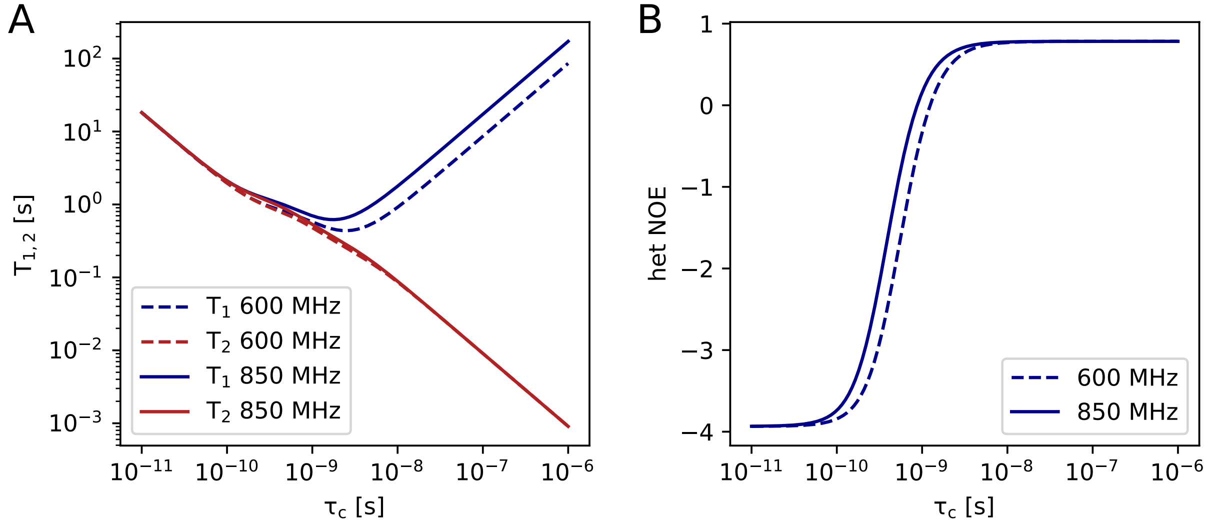

Figure S17: Dependence of T_1, T_2 and hetNOE on the correlation times τ_c (timescales of molecular motion). The theoretical spin relaxation times (**A**) and hetNOE values (**B**) were computed using the Redfield equations[13,50] assuming field strengths of 600 and 850 MHz.

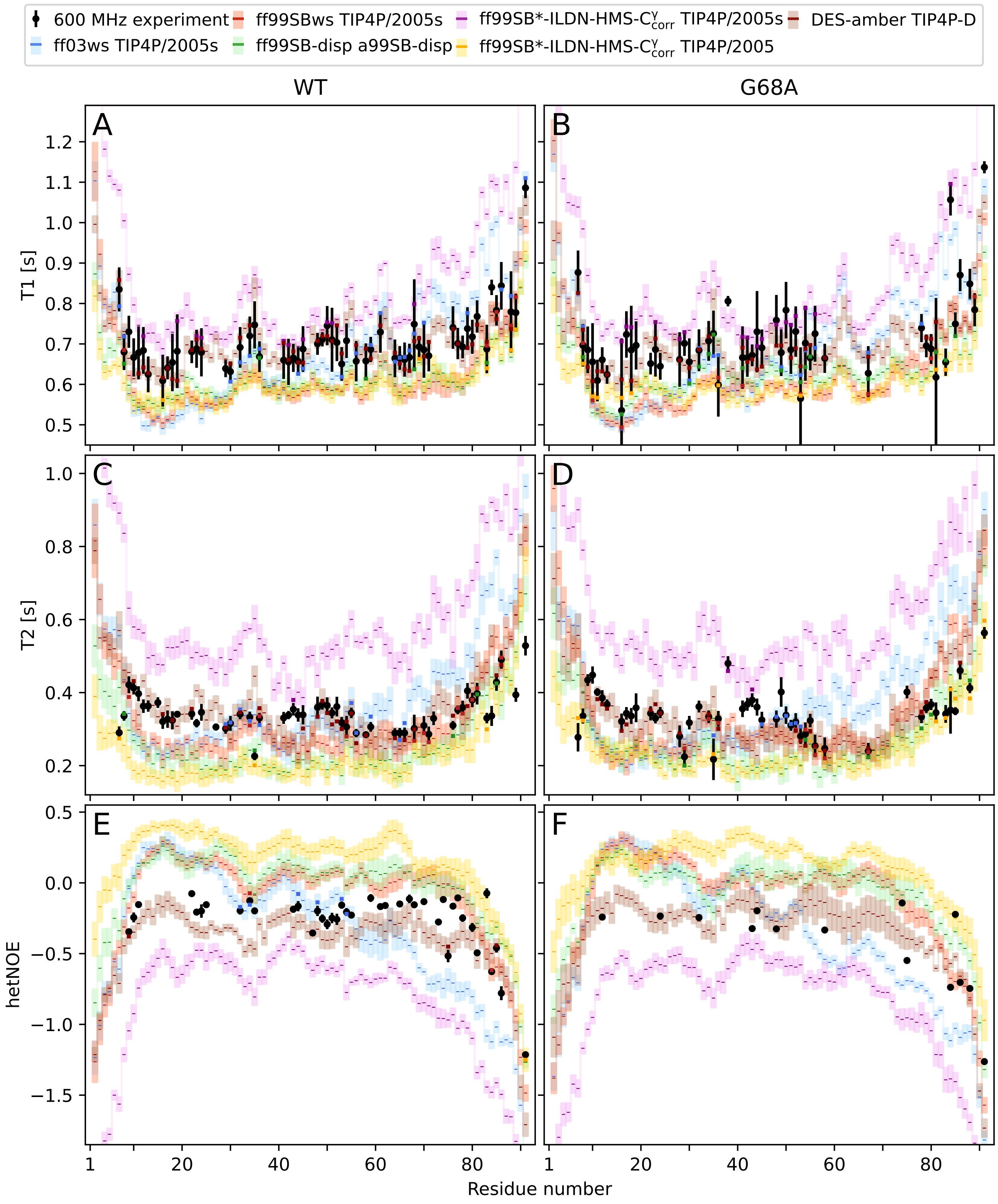

Figure S18: NMR relaxation parameters T_1 (**A**,**B**), T_2 (**C**,**D**) and hetNOE (**E**,**F**) computed from simulation of COR15A WT (**A**,**C**,**D**) and G68A (**B**,**D**,**F**) in comparison to experimental data measured at a field strength of 600 MHz. The relaxation parameters per residue were computed from MD simulations using the MD systems ff03ws, ff99SBws, ff99SB-disp, ff99SB*-ILDN-HMS-C^{γ}_{corr} in combination with the water models TIP4P/2005 and TIP4P/2005s and DES-amber and are shown in comparison to the experimental data (black dots). The SEM is indicated in lighter colors for the computed data and error bars indicate the experimental error.

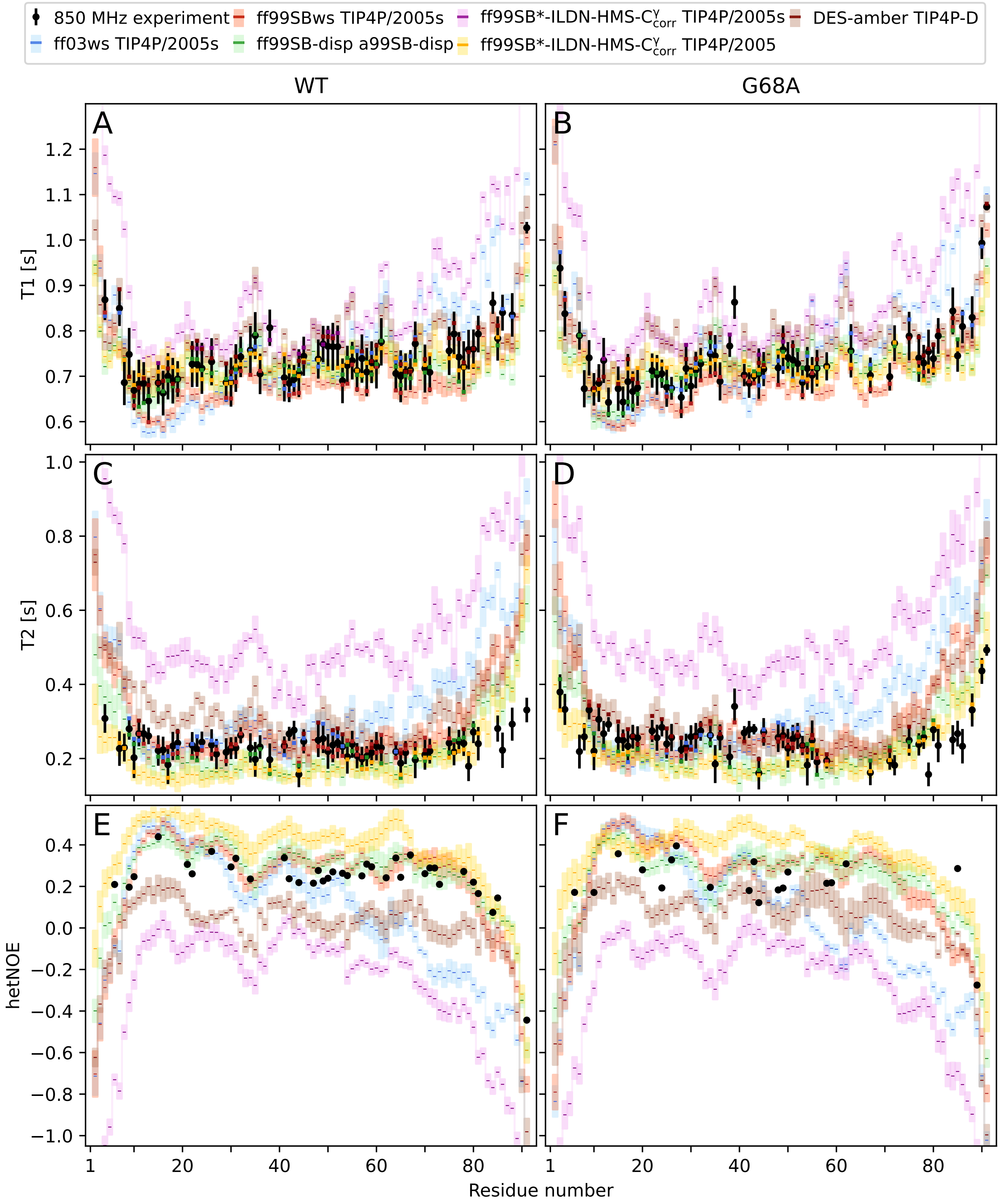

Figure S19: NMR relaxation parameters T_1 (**A**,**B**), T_2 (**C**,**D**) and hetNOE (**E**,**F**) computed from simulation of COR15A WT (**A**,**C**,**D**) and G68A (**B**,**D**,**F**) in comparison to experimental data measured at a field strength of 850 MHz. The relaxation parameters per residue were computed from MD simulations using the MD systems ff03ws, ff99SBws, ff99SB-disp, ff99SB*-ILDN-HMS-C^{γ}_{corr} in combination with the water models TIP4P/2005 and TIP4P/2005s and DES-amber and are shown in comparison to the experimental data (black dots). The SEM is indicated in lighter colors for the computed data and error bars indicate the experimental error.

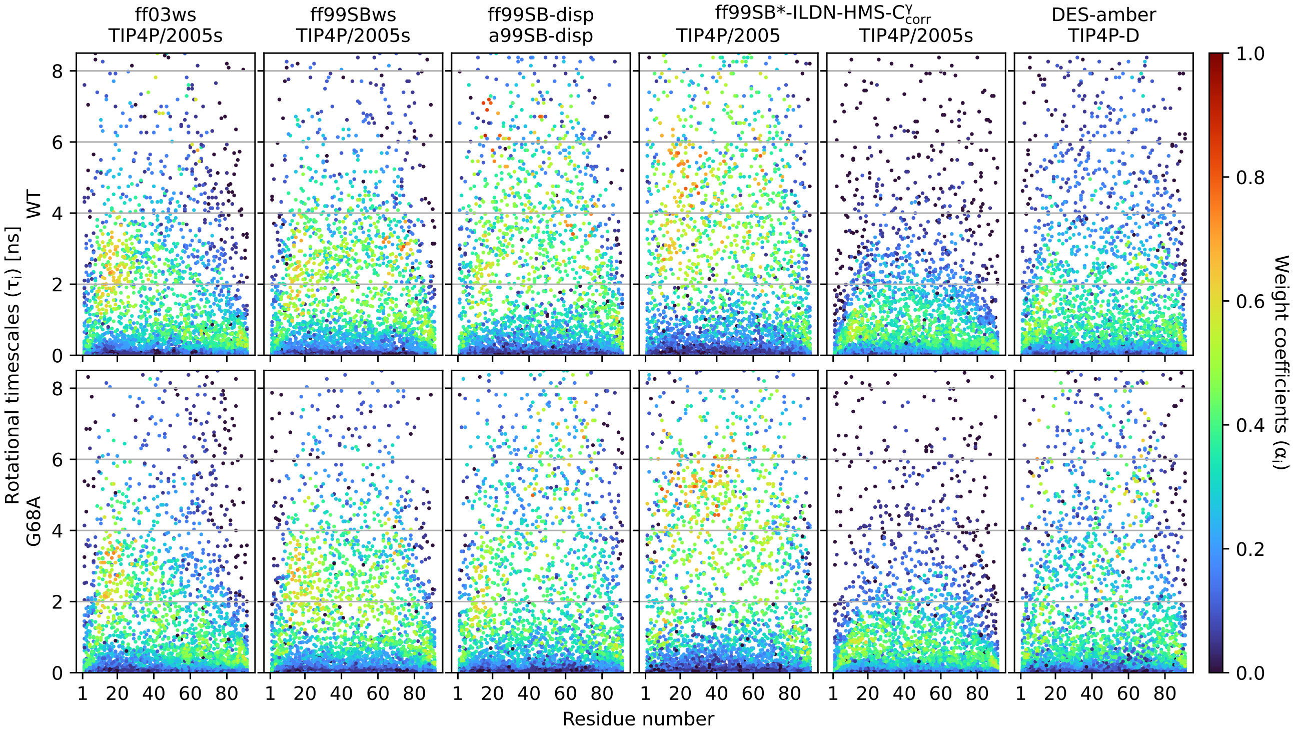

Figure S20: Rotational timescales (τ_i) calculated according to Eq 11 from MD simulations of COR15A WT and G68A with related weight coefficient $\alpha_i > 0$ are shown per residue. The corresponding weight coefficient α_i of every data point is depicted by a color gradient. Demonstrated are the individual results of 10 simulations with the MD systems ff03ws, ff99SBws, ff99SB-disp, ff99SB*-ILDN-HMS-C$^{\gamma}_{\mathrm{corr}}$ in combination with the water models TIP4P/2005 and TIP4P/2005s and DES-amber in linear presentation.

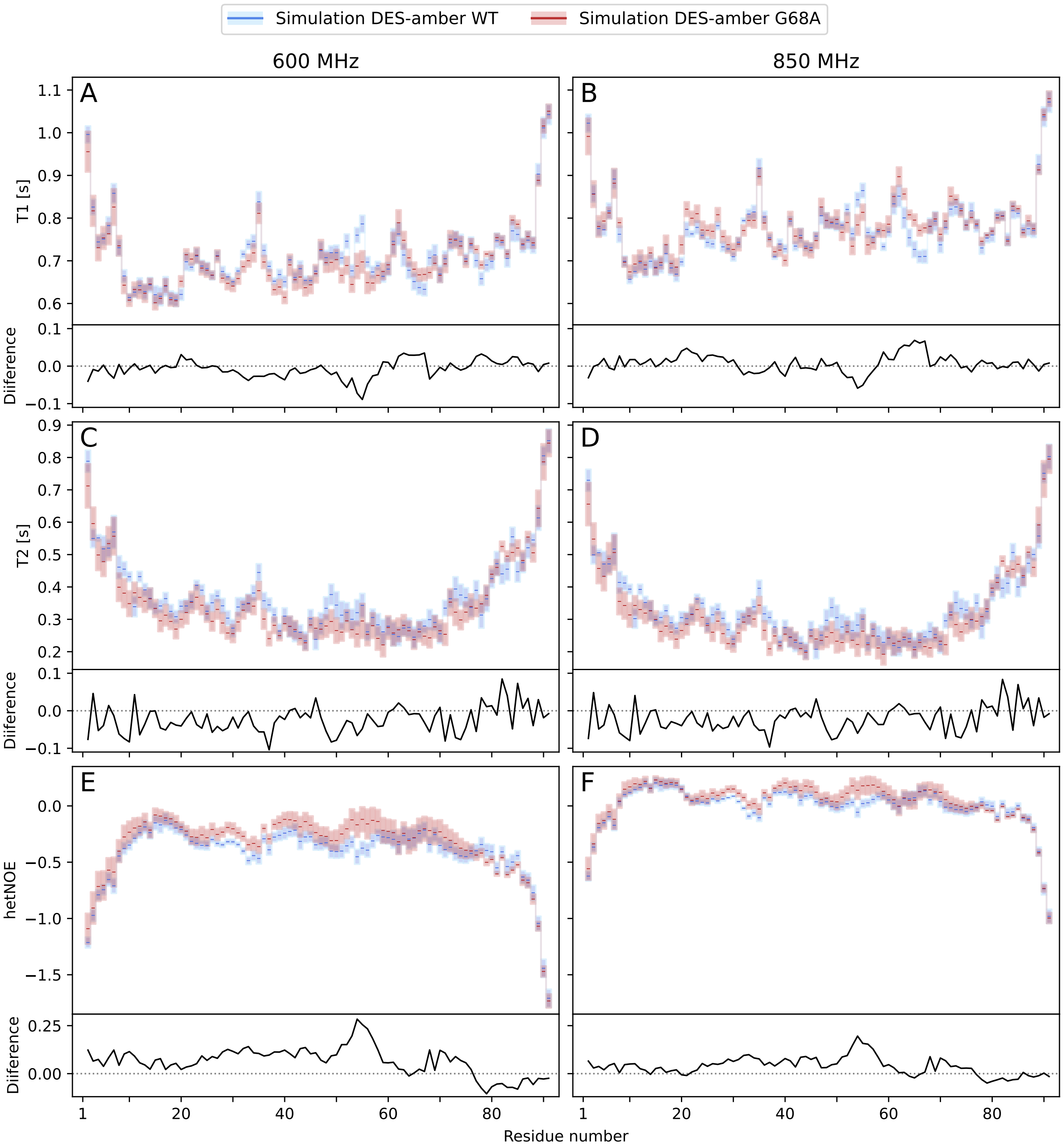

Figure S21: NMR relaxation parameters T_1 (**A**,**B**), T_2 (**C**,**D**) and hetNOE (**E**,**F**) computed from DES-amber simulations of COR15A WT and COR15A G68A at a field strength of 600 MHz (**A**,**C**,**D**) and 850 MHz (**B**,**D**,**F**) are shown in the upper panel of each subplot. The lower panels indicate the difference between the G68A mutant and the COR15A WT data. The SEM is indicated in lighter colors for the computed data.

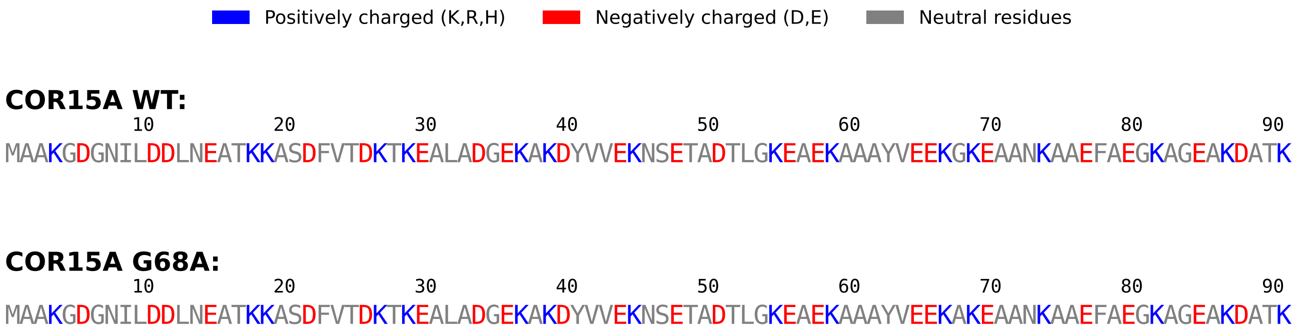

Figure S22: Amino acid sequences of COR15A WT and G68A, with residues colored by charge (blue: positive; red: negative; gray: neutral).

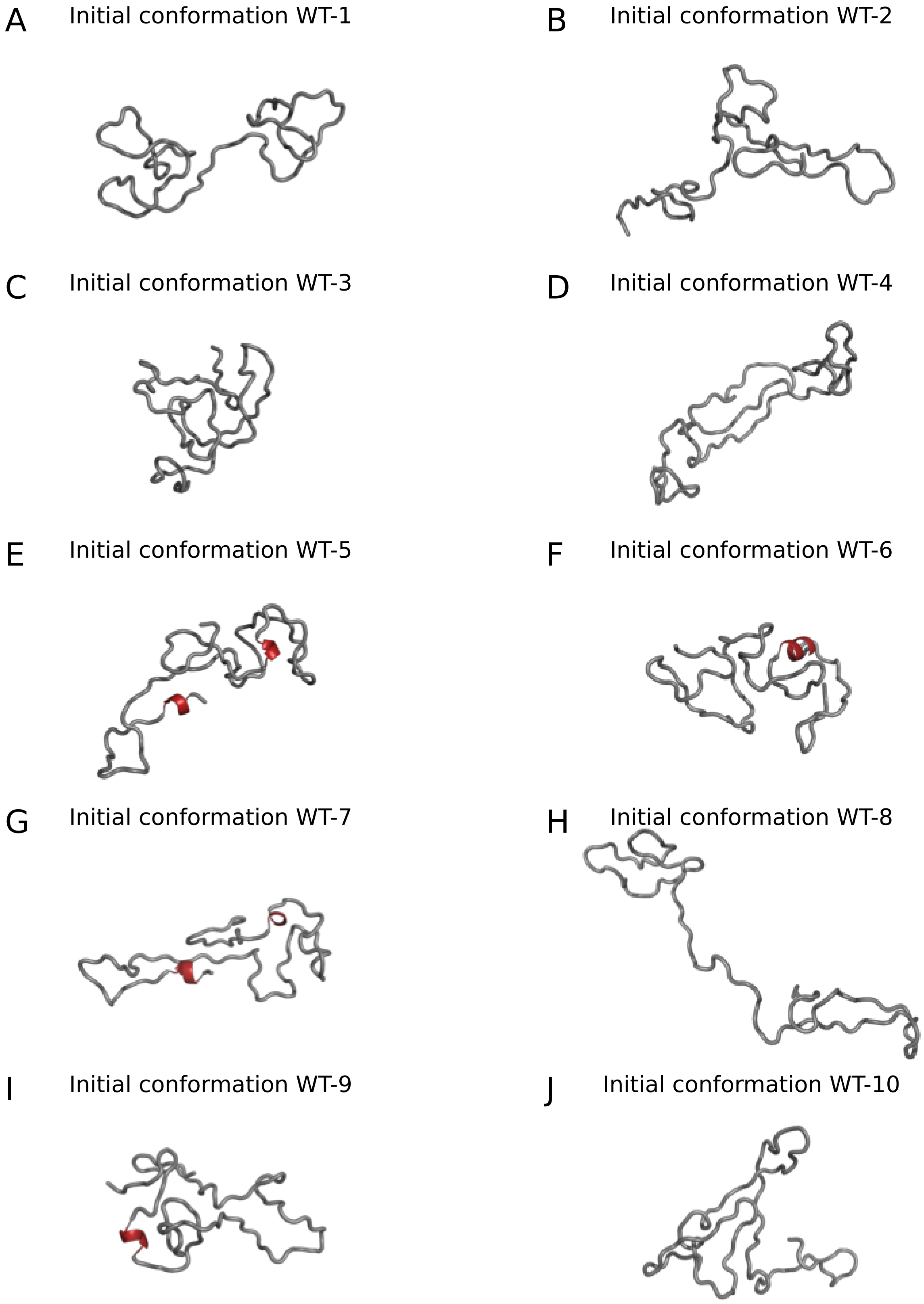

Figure S23: The ten structures from structure modelling constituting the initial conformational ensemble of COR15A WT are represented in cartoon representation. Disordered regions (random coil) are illustrated in grey, α-helices are indicated in red. The chosen structures represent the centromeres of the corresponding trajectories. Size of images is scaled.

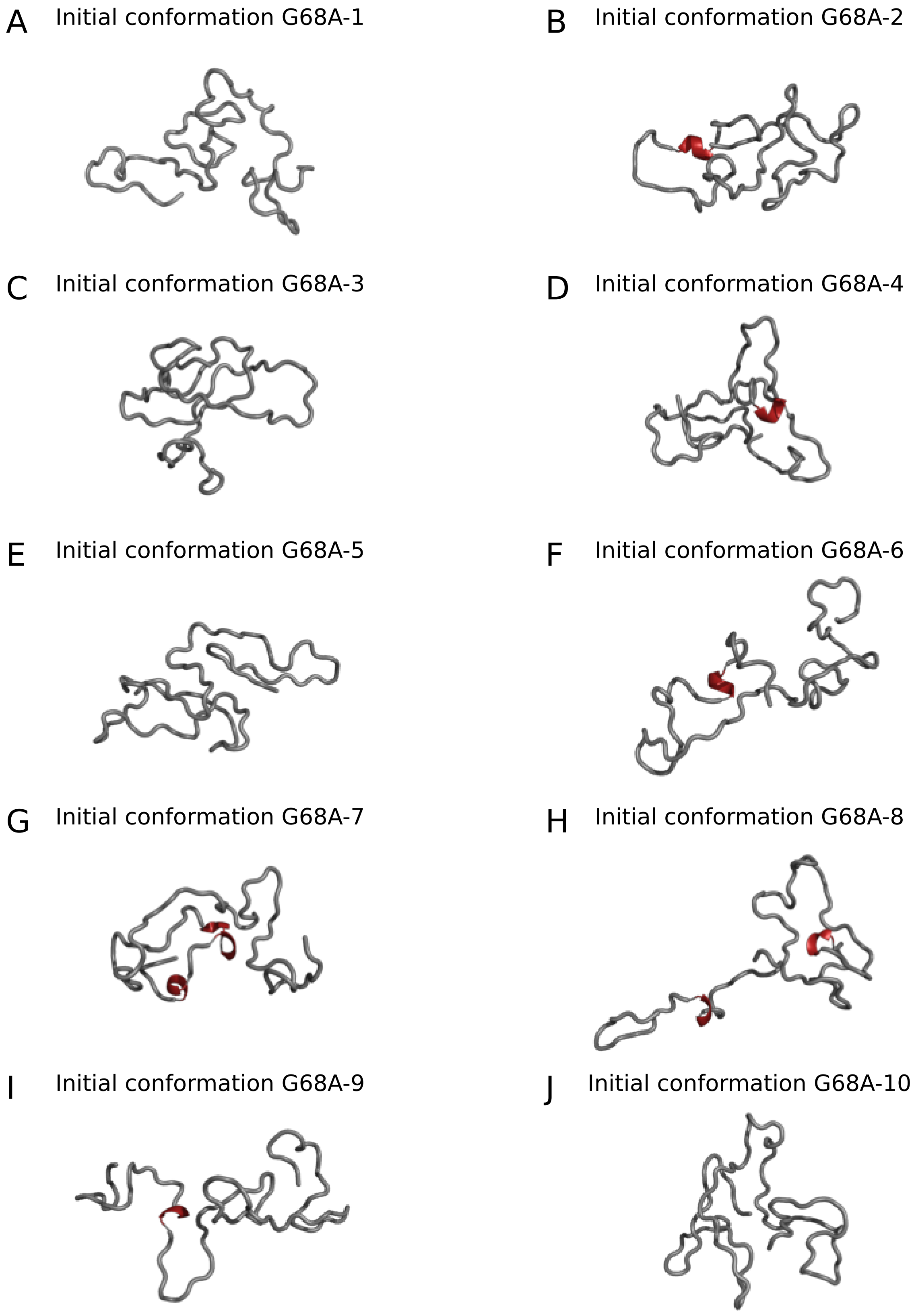

Figure S24: The ten structures from structure modelling constituting the initial conformational ensemble of G68A are represented in cartoon representation. Disordered regions (random coil) are illustrated in grey, α-helices are indicated in red. The chosen structures represent the centromeres of the corresponding trajectories. Size of images is scaled.

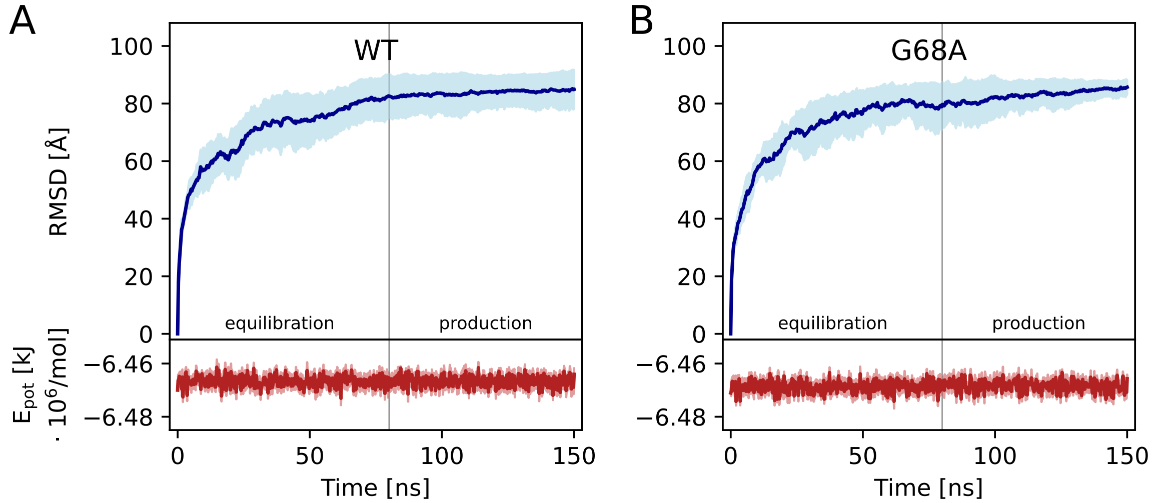

Figure S25: Root mean square deviation (RMSD) and potential energy (E_{pot}) during simulations for the initial IDP ensemble generation of COR15A WT (**A**) and G68A (**B**). The upper figure in each subplot shows the development of the RMSD averaged over all initial simulations based on the backbone atoms of the protein while the corresponding standard deviations are marked in lighter colours. The lower figure in each subplot represents the averaged potential energy (E_{pot}) for the corresponding simulation system in the upper subplot.

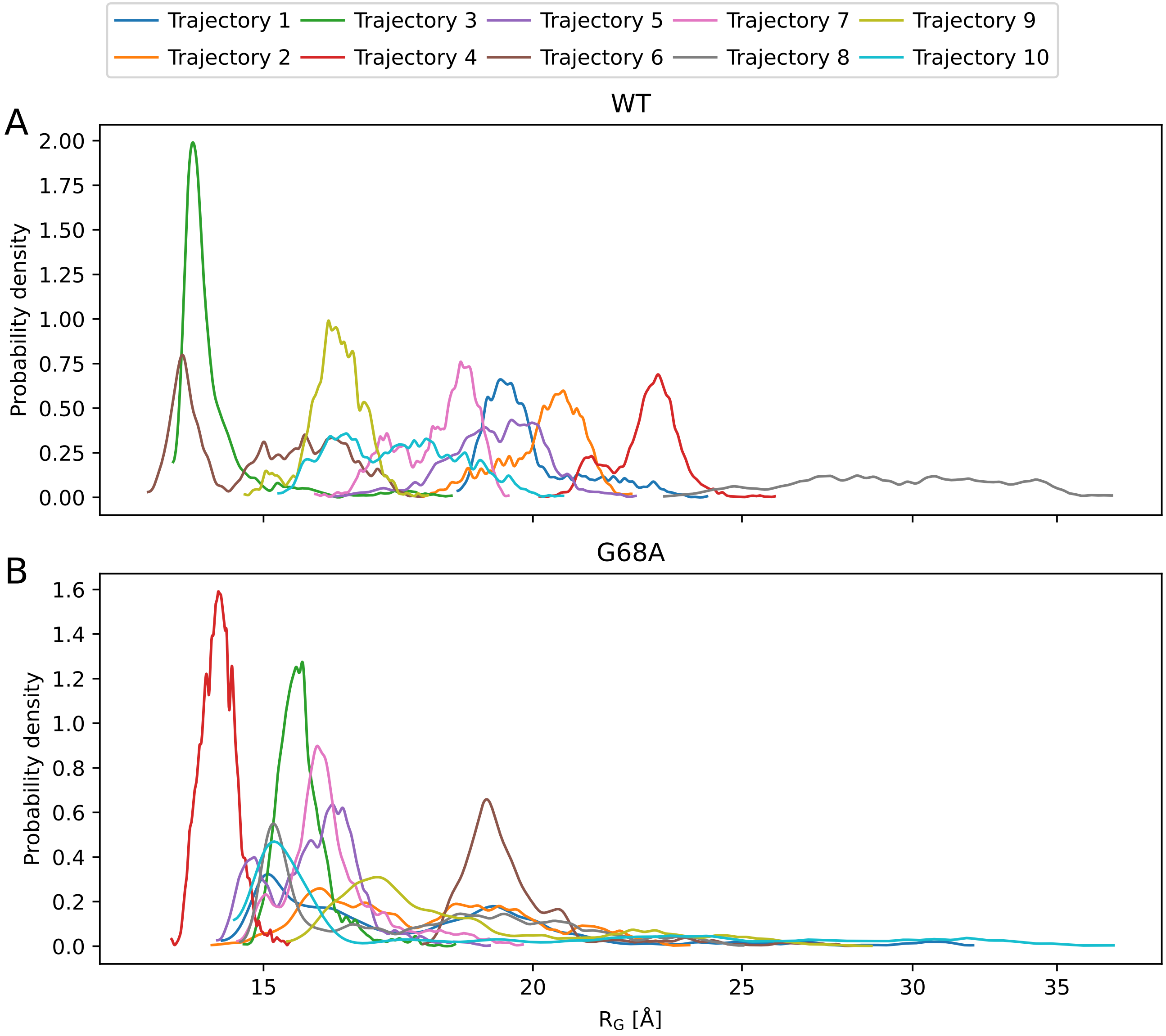

Figure S26: Probability density per trajectory of the radius of gyration (R_G) from ensembles generated during the initial structure modelling of COR15A WT (**A**) and G68A (**B**), respectively. All probability density distributions are represented by 500 bins and the resulting curves were slightly smoothed using a gaussian filter implemented in the python package `scipy`[8] for clearer representation.

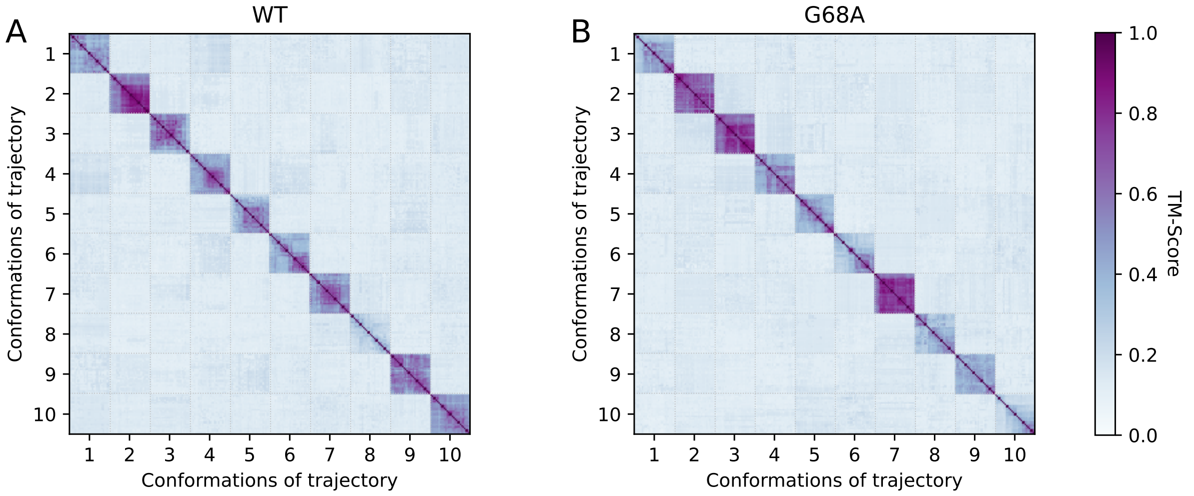

Figure S27: Pairwise distance matrices of initial COR15A WT (**A**) and G68A (**B**) models for all the 250 extracted conformations of each initial structure modelling are shown as heat map for visualizing the similarity of the structures. Axis numbering refers to the ten trajectories which each comprise 25 corresponding models represented as single dots. The distances (TM-Score[14]) were determined using the MaxCluster[15] program and are indicated by a white-blue-purple colour code referring to low (TM = 0) to high (TM = 1) similarity.

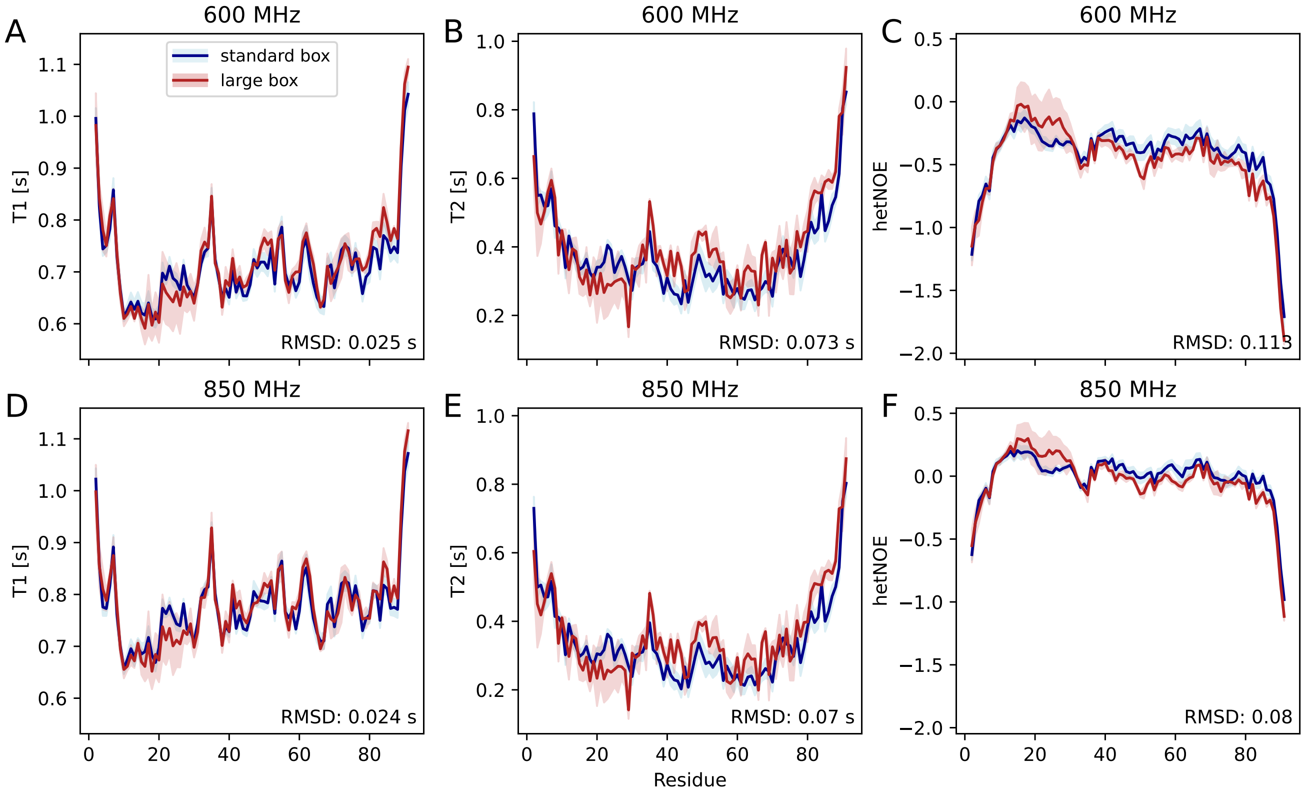

Figure S28: NMR relaxation parameters T_1 (**A**, **D**), T_2 (**B**, **E**), and hetNOE (**C**, **F**) computed from DES-amber simulations of COR15A WT at field strengths of 600 MHz (**A**–**C**) and 850 MHz (**D**–**F**) are shown for simulations run in the standard box (diameter: 120 Å) and a significantly larger box (diameter: 270 Å). The RMSD between the two curves in each subplot is indicated. Shading represents standard error of the mean (SEM, n=10 for the standard and n=3 for the larger box).

References

(1) Roy, A.; Kucukural, A.; Zhang, Y. I-TASSER: a unified platform for automated protein structure and function prediction. *Nature Protocols* **2010**, *5*, 725–738.

(2) Dey, S.; MacAinsh, M.; Zhou, H.-X. Sequence-Dependent Backbone Dynamics of Intrinsically Disordered Proteins. *Journal of Chemical Theory and Computation* **2022**, *18*, 6310–6323.

(3) Yu, L.; Brüschweiler, R. Quantitative prediction of ensemble dynamics, shapes and contact propensities of intrinsically disordered proteins. *PLOS Computational Biology* **2022**, *18*, e1010036.

(4) Mészáros, B.; Erdős, G.; Dosztányi, Z. IUPred2A: context-dependent prediction of protein disorder as a function of redox state and protein binding. *Nucleic Acids Research* **2018**, *46*, W329–W337.

(5) Shou, K.; Bremer, A.; Rindfleisch, T.; Knox-Brown, P.; Hirai, M.; Rekas, A.; Garvey, C. J.; Hincha, D. K.; Stadler, A. M.; Thalhammer, A. Conformational selection of the intrinsically disordered plant stress protein COR15A in response to solution osmolarity–an X-ray and light scattering study. *Physical Chemistry Chemical Physics* **2019**, *21*, 18727–18740.

(6) Dima, R. I.; Thirumalai, D. Asymmetry in the Shapes of Folded and Denatured States of Proteins. *The Journal of Physical Chemistry B* **2004**, *108*, 6564–6570.

(7) Hofmann, H.; Soranno, A.; Borgia, A.; Gast, K.; Nettels, D.; Schuler, B. Polymer scaling laws of unfolded and intrinsically disordered proteins quantified with single-molecule spectroscopy. *Proceedings of the National Academy of Sciences* **2012**, *109*, 16155–16160.

(8) Virtanen, P.; Gommers, R.; Oliphant, T. E.; Haberland, M.; Reddy, T.; Cournapeau, D.; Burovski, E.; Peterson, P.; Weckesser, W.; Bright, J.; van der Walt, S. J.; Brett, M.; Wilson, J.; Jarrod Millman, K.; Mayorov, N.; Nelson, A. R. J.; Jones, E.; Kern, R.; Larson, E.; Carey, C.; Polat, İ.; Feng, Y.; Moore, E. W.; Vand erPlas, J.; Laxalde, D.; Perktold, J.; Cimrman, R.; Henriksen, I.; Quintero, E. A.; Harris, C. R.; Archibald, A. M.; Ribeiro, A. H.; Pedregosa, F.; van Mulbregt, P. SciPy 1.0: Fundamental Algorithms for Scientific Computing in Python. *Nature Methods* **2020**, *17*, 261–272.

(9) Touw, W. G.; Baakman, C.; Black, J.; Te Beek, T. A.; Krieger, E.; Joosten, R. P.; Vriend, G. A series of PDB-related databanks for everyday needs. *Nucleic Acids Research* **2015**, *43*, D364–D368.

(10) Kabsch, W.; Sander, C. Dictionary of protein secondary structure: pattern recognition of hydrogen-bonded and geometrical features. *Biopolymers: Original Research on Biomolecules* **1983**, *22*, 2577–2637.

(11) Sowemimo, O. T.; Knox-Brown, P.; Borcherds, W.; Rindfleisch, T.; Thalhammer, A.; Daughdrill, G. W. Conserved Glycines Control Disorder and Function in the Cold-Regulated Protein, COR15A. *Biomolecules* **2019**, *9*, 84.

(12) Shen, Y.; Bax, A. SPARTA+: a modest improvement in empirical NMR chemical shift prediction by means of an artificial neural network. *Journal of Biomolecular NMR* **2010**, *48*, 13–22.

(13) Ollila, O. S.; Heikkinen, H. A.; Iwaï, H. Rotational Dynamics of Proteins from Spin Relaxation Times and Molecular Dynamics Simulations. *The Journal of Physical Chemistry B* **2018**, *122*, 6559–6569.

(14) Zhang, Y.; Skolnick, J. Scoring function for automated assessment of protein structure template quality. *Proteins: Structure, Function, and Bioinformatics* **2004**, *57*, 702–710.

(15) Herbert, A.; Sternberg, M. MaxCluster–A tool for Protein Structure Comparison and Clustering. 2014. *URL: http://www. sbg. bio. ic. ac. uk/˜ maxcluster* **2008**,
